# A multi-omics approach elucidates the link between artificial food colorings and common cancers

**DOI:** 10.3389/fnut.2026.1743416

**Published:** 2026-02-05

**Authors:** Xiang Feng, Biyuan Luo, Mengge Ding, Xianling Liu

**Affiliations:** 1 Department of Oncology, The Second Xiangya Hospital of Central South University, Changsha, China; 2 Department of Oncology, Guilin Hospital of the Second Xiangya Hospital, Central South University, Guilin, China

**Keywords:** AFCs, artificial food colorings, cancer, machine learning, network toxicology, prognosis

## Abstract

**Background:**

Artificial food colorings (AFCs) are widely used, yet their potential links to cancer remain unclear. We investigated associations between commonly used AFCs and cancer-related molecular networks and prognosis.

**Methods:**

AFCs-related targets were collected from CTD, ChEMBL, SEA, and TargetNet, and cancer-related targets from GeneCards, OMIM, and CTD. Overlapping targets were subjected to STRING-based PPI analysis and Cytoscape visualization, followed by GO/KEGG enrichment. Core targets were evaluated for differential expression in GEO datasets of non-small cell lung cancer (NSCLC), colon adenocarcinoma (COAD), gastric cancer (GC), and breast cancer (BRCA), with GSEA for pathway characterization. Expression patterns were examined using GEPIA2. TCGA transcriptomic and clinical data were used to construct prognostic models via univariate Cox regression, LASSO selection, and multivariate Cox regression. Key genes were assessed using the Human Protein Atlas (HPA) and qPCR, and *in vivo* experiments evaluated tumor growth under AFCs exposure.

**Results:**

Four high-exposure AFCs were analyzed. We identified 108 shared AFCs–cancer targets and prioritized 50 core targets. Enrichment analyses highlighted cancer-relevant functional themes, including cell-cycle regulation (cyclin-dependent protein kinase holoenzyme complex) and oncogenic signaling (PI3K–Akt pathway). Multiple core targets were dysregulated in GEO tumor datasets, and GSEA identified consistently enriched pathways across cancer types. TCGA-derived signatures stratified patients into distinct risk groups with significantly different overall survival. HPA supported protein-level differences for selected targets, qPCR indicated that Allura Red AC or Tartrazine modulated prognostic gene expression in cancer cell lines, and AFCs exposure was associated with accelerated LLC tumor growth in mice.

**Conclusion:**

This integrative analysis suggests that commonly used AFCs may be associated with cancer-related molecular networks and adverse prognosis in NSCLC, COAD, GC, and BRCA, informing future safety evaluation and regulation.

## 1 Introduction

Artificial food colorings (AFCs) are chemically synthesized additives widely incorporated into processed foods to improve product appearance and palatability, thereby enhancing consumer acceptance (1, 2). Although their use is regulated by national and international agencies, persistent concerns remain regarding their safety (1). Emerging evidence has associated AFCs exposure with multiple adverse health outcomes, including attention-deficit/hyperactivity disorder (ADHD), neurotoxicity, intestinal inflammation, and carcinogenic potential (2–4). Given their extensive consumption, clarifying the health effects of AFCs in humans remains an important public health priority.

The use of food colorants can be traced back to ancient Egypt, where they were applied in the preparation of candies and wines, as color was regarded as an indicator of food quality (5). This early practice subsequently led to the incorporation of various natural pigments in food processing (5). In 1856, the synthesis of the first artificial dye, mauveine, marked the beginning of the use of AFCs (5). Compared with natural colorants, AFCs exhibit several advantages, including lower production costs, greater stability, and improved coloring efficiency, which have contributed to their widespread use in modern food manufacturing (6). In recent years, increasing awareness of the potential health risks associated with AFCs has prompted governments worldwide to introduce stricter regulatory measures (1). For example, the European Union implemented Regulation (EC) No 1333/2008 on food additives, under which only 15 AFCs are authorized for use in food, with clearly defined categories and maximum permitted levels (7). In addition, foods containing any of six specific AFCs are required to carry a warning label indicating a possible association with hyperactivity in children (7). In the United States, the U.S. Food and Drug Administration (FDA) authorizes nine AFCs for use in food, as specified in Title 21 of the Code of Federal Regulations (8), while 11 AFCs are currently permitted in China (9). The heterogeneity of national regulatory frameworks has resulted in differences in the approval and use of AFCs across regions, underscoring the need for strengthened global regulation and comprehensive safety evaluation.

A growing body of evidence has drawn attention to the potential health risks associated with AFCs. Among the reported adverse effects, neurotoxicity has been frequently described. A large-scale cohort study reported that higher dietary intake of AFCs was associated with an increased risk of ADHD (10). Mechanistically, AFCs have been proposed to contribute to hyperactivity and learning difficulties in children by modulating the expression of NR2A and NR2B, which are implicated in synaptic signaling and neurodevelopment (3). In addition, AFCs may affect neurodevelopment and neurological function through interactions with hormone receptors and by influencing gene pathways related to oxidative stress and inflammatory responses (11). Notably, erythrosine B has been reported to promote amyloid-β aggregation, suggesting a potential contribution to Alzheimer’s disease-related pathology and raising concern regarding AFCs-induced neurofunctional impairment (12). Beyond the nervous system, AFCs have been implicated in intestinal inflammation. Experimental evidence indicates that AFCs exposure can induce inflammatory bowel disease (IBD)-like colitis, potentially through upregulation of the pro-inflammatory cytokine IL-23 (4). AFCs have also been linked to gut microbiota dysbiosis, with alterations in microbial composition and activity that may promote a pro-inflammatory milieu, impair barrier integrity, and exacerbate immune dysregulation under pathological conditions (13). Moreover, Allura Red AC exposure has been associated with DNA damage in murine colonic tissue, accompanied by genetic alterations and local changes in the gastrointestinal microenvironment (14). Collectively, these findings suggest that AFCs may participate in multiple pathological processes, including inflammation, chromatin damage, and allergic responses (2, 6).

**Figure 1:**
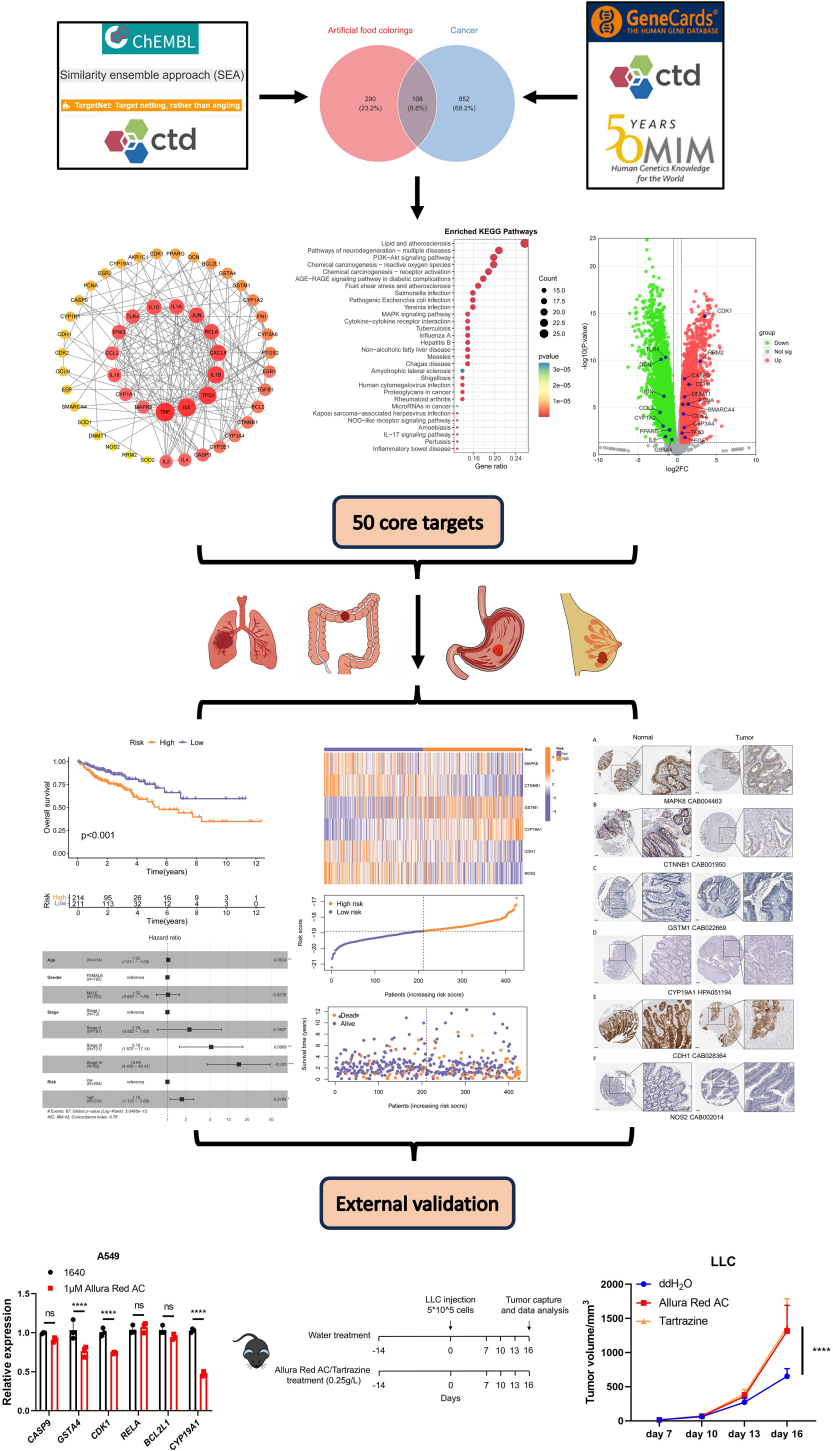
Schematic diagram of the study design.

The association between AFCs and cancer remains controversial and has not been thoroughly characterized. According to global cancer burden statistics, lung, breast, colorectal, and gastric cancers rank among the most common malignancies worldwide and account for a substantial proportion of cancer-related deaths (15). In this study, we comprehensively evaluated the relationship between AFCs and these four cancers by integrating database mining, machine learning, network toxicology, TCGA-based prognostic analyses, and external validation. Our results may help inform regulatory decision-making and support evidence-based safety evaluation of AFCs. Using systematic data mining and integrative analyses, we identified 50 core genes potentially linking AFCs to cancer-related pathways. The overall study workflow is presented in Figure 1.

## 2 Methods

### 2.1 Selection of artificial food colorings (AFCs) and collection of their targets

Regulatory approaches to AFCs vary across regions, reflecting differences in national and regional legislation. To date, population-level dietary exposure to AFCs has been evaluated mainly in the European Union and the United States, whereas comparable assessments are unavailable for most other countries (16). Overall, existing evidence suggests that dietary exposure to AFCs generally remains below the acceptable daily intake (ADI) in both the European Union and the United States (16, 17). In the United States, AFCs production has increased steadily since the mid-1950s (18). An analysis of approximately 810 food products in 2016 reported that more than half contained artificial colorants. The four most frequently detected AFCs were Allura Red AC (29.8% of products), Brilliant Blue FCF (24.2%), Tartrazine (20.5%), and Sunset Yellow FCF (19.5%); collectively, these accounted for over 90% of the AFCs occurrences identified (19). Subsequent exposure assessments in children, pregnant women, and women of childbearing age reported intake patterns consistent with the 2016 findings, with U.S. children showing higher intake than adults (20). Multiple estimates further indicate that population exposure in the United States remains below ADI values established by FDA and the Joint FAO/WHO Expert Committee on Food Additives (JECFA) (17, 20). Notably, short-term intake exceeding the ADI for FD&C Red No. 3 has been documented in subsets of children and pregnant women, and has been associated with neurobehavioral concerns in children (20). However, FD&C Red No. 3 was identified in only 1.9% of products in the 2016 survey, substantially lower than other high-use AFCs (19). In addition, its use is increasingly restricted; FDA-certified volumes of FD&C Red No. 3 in fiscal year 2025 were markedly lower than those of other widely used colorants (Supplementary Table 1). Accordingly, subsequent analyses were restricted to the major high-exposure AFCs identified in the U.S. survey, and FD&C Red No. 3 was excluded.

Four widely used AFCs (Allura Red AC, Brilliant Blue FCF, Tartrazine, and Sunset Yellow FCF) were selected for subsequent analyses. Key information for these AFCs, including acceptable daily intake (ADI) values and regulatory status in the United States, the European Union, and China, is summarized in Table 1 and Supplementary Table 2. Potential targets were retrieved from the CTD^1^ using each AFCs name as a keyword. Chemical structures (SMILES and SDF) were obtained from PubChem.^2^ In addition, target prediction/screening was conducted using ChEMBL,^3^ SEA,^4^ and TargetNet,^5^ restricting the organism to *Homo sapiens*. The following thresholds were applied: SEA (Z-score > 10) and TargetNet (probability > 0.5). Targets obtained from all sources were integrated. For each AFCs, targets were first merged and deduplicated to generate a compound-specific unique target set. These four sets were then combined to generate the overall target set for downstream analyses. Target set overlap and merging were performed in R using the “ggvenn” package (v0.1.10).

**Table 1:** Characteristics of the four commonly used AFCs.

Number	Molecular	Formula	CAS ID	MW(g/moL)
1	Allura Red AC	C18H14N2Na2O8S2	25956-17-6	496.43
2	Brilliant Blue FCF	C37H34N2Na2O9S3	3844-45-9	792.85
3	Tartrazine	C16H9N4Na3O9S2	1934-21-0	534.36
4	Sunset Yellow FCF	C16H10N2Na2O7S2	2,783-94-0	452.37

### 2.2 Obtaining cancer-related targets

Cancer-associated targets were collected from GeneCards,^6^ OMIM,^7^ and the CTD using the keyword “cancer” (see footnote 1). Searches were restricted to *Homo sapiens*. The following filters were applied: GeneCards relevance score > 20 and CTD inference score > 100. Targets from the three sources were merged and deduplicated based on gene symbols to generate a non-redundant cancer target set. Overlap and target set merging were performed in R using the “ggvenn” package (v0.1.10).

### 2.3 AFCs-cancer targets acquisition and PPI network construction

The intersecting AFCs–cancer targets were identified in R using the “ggvenn” package (v0.1.10). The resulting gene list was submitted to the STRING database for protein–protein interaction (PPI) network construction, with the organism restricted to *Homo sapiens* and the confidence score threshold set to 0.9. After removal of isolated nodes, the PPI network was generated. Network data were exported in TSV format and imported into Cytoscape (v3.8.0) for visualization. Network topology parameters were calculated using the built-in “Analyze Network” function in Cytoscape. Node importance was evaluated based on degree, betweenness centrality, and closeness centrality, with higher values indicating greater topological relevance.

### 2.4 GO and KEGG analysis

Gene Ontology (GO) and Kyoto Encyclopedia of Genes and Genomes (KEGG) pathway enrichment analyses were conducted for the AFCs–cancer targets using the R package “clusterProfiler” (v4.12.6), and visualized with “enrichplot” (v1.24.4). GO enrichment was performed across the biological process (BP), cellular component (CC), and molecular function (MF) domains. For each term/pathway, both raw *p*-values and adjusted *p*-values (p.adjust) were calculated and reported; however, statistical significance was defined based on raw p values, with *p* < 0.05 considered significant. Enriched GO terms were ranked by *p*-value (ascending) and displayed as bubble plots. For KEGG analysis, the top 30 significantly enriched pathways ranked by *p*-value were selected for visualization.

### 2.5 Retrieve data from GEO database and TCGA database

Gene expression microarray datasets for non-small cell lung cancer (NSCLC), colon adenocarcinoma (COAD), gastric cancer (GC), and breast cancer (BRCA) were downloaded from the Gene Expression Omnibus (GEO) database: GSE74706 (NSCLC), GSE68468 (COAD), GSE19826 (GC), and GSE22820 (BRCA). Dataset details are provided in Table 2. In addition, transcriptomic profiles and corresponding clinical information for survival analyses were obtained from The Cancer Genome Atlas (TCGA), including TCGA-LUAD and TCGA-LUSC for NSCLC, TCGA-COAD for COAD, TCGA-STAD for GC, and TCGA-BRCA for breast cancer. The sample sizes were 1,162 (NSCLC), 254 (COAD), 448 (GC), and 1,231 (BRCA).

**Table 2:** GEO datasets.

GEO accession	Platform	Tissue	Normal	Tumor	Reference
GSE74706	GPL13497	Lung	18	18	27197161
GSE68468	GPL96	Colon	55	195	16489013
GSE19826	GPL570	Stomach	12	12	21132402
GSE22820	GPL6480	Breast	10	176	21356353

### 2.6 Screening of differentially expressed genes (DEGs) in GEO datasets

The four GEO microarray datasets (GSE74706, GSE19826, GSE68468, and GSE22820) were preprocessed and analyzed for differential expression using the R package “limma” (v3.60.6). Raw expression data were background-corrected and normalized prior to DEG analysis, and probe intensities were summarized at the gene level. For genes represented by multiple probes, the probe with the highest mean expression across all samples was retained. Differentially expressed genes (DEGs) were defined using a raw p value < 0.05 and |log2 fold change (log2FC) | > 0.5, and were classified as upregulated or downregulated accordingly. DEG lists were uploaded to the CNSknowall platform^8^ to generate volcano plots.

### 2.7 GSEA analysis in GEO datasets

Genes identified in Section 2.6 were further filtered using raw *p* < 0.05 and |log2FC| > 0.01. The retained genes were ranked by log2FC (descending) to generate a preranked list for Gene Set Enrichment Analysis (GSEA). The relaxed fold-change threshold was used to retain sufficient genes for stable enrichment analysis. GSEA was performed using “clusterProfiler” (v4.12.6) with annotation support from “org.Hs.eg.db” (v3.19.1), and results were visualized using “enrichplot” (v1.24.4). The top 10 enriched KEGG pathways were selected for presentation.

### 2.8 Common cancer analysis of AFCs-cancer targets

Four common cancers were included for downstream analyses, including NSCLC, COAD, GC, and BRCA. The AFCs–cancer core targets were queried in the GEPIA2 database to assess their expression profiles across NSCLC, COAD, GC, and BRCA. The resulting expression outputs were then uploaded to the CNSknowall platform for visualization (see footnote 8).

### 2.9 Construction and evaluation of cancer risk prognostic model

To identify prognostic genes among AFCs-associated cancer-related targets (AFCs-cancer core targets) in NSCLC, COAD, GC, and BRCA, transcriptomic profiles and overall survival data were obtained from TCGA database for each cancer type. Univariate Cox proportional hazards regression was first performed for all AFCs–cancer core targets using the R package “survival” (v3.8–3). Genes with a raw *p*-value < 0.05 were considered prognosis-related and retained for subsequent analyses. The univariate Cox regression results were visualized as forest plots using the “survminer” package (v0.5.0). To reduce model complexity and prevent overfitting, Least Absolute Shrinkage and Selection Operator (LASSO) regression was applied to further select candidate prognostic genes using the “glmnet” package (v4.1–10). Genes retained after LASSO regression were subsequently entered into a multivariate Cox regression model constructed with the “survival” package (v3.8–3) to establish the prognostic risk model. Model interpretability was assessed using SHapley Additive exPlanations (SHAP) analysis, implemented with the “kernelshap” (v0.9.0) and “shapviz” (v0.10.2) packages. The risk score (RS) for each patient was calculated according to the following formula: RS = *Σ* (Coef_gene × Exp_gene), where Coef_gene represents the regression coefficient of each prognostic gene derived from the multivariate Cox model, and Exp_gene denotes the corresponding gene expression level. Patients were stratified into high- and low-risk groups based on the median RS. Kaplan–Meier (KM) survival analysis was performed using the “survival” (v3.8–3) and “survminer” (v0.5.0) packages to compare overall survival between the high- and low-risk groups, and survival curves were generated. A raw *p*-value < 0.05 was considered statistically significant. The predictive performance of the prognostic model was further evaluated using time-dependent receiver operating characteristic (ROC) analysis with the “timeROC” package (v0.4), and the area under the curve (AUC) was calculated for 1-, 3-, and 5-year overall survival. In addition, the “pheatmap” package (v1.0.12) was used to visualize patient survival status, expression patterns of the prognostic genes, and corresponding risk scores.

### 2.10 Protein validation in normal and cancer tissues

Protein-level validation of key prognostic targets was performed using the Human Protein Atlas (HPA) database.^9^ Protein expression patterns of the selected targets were examined in NSCLC, COAD, GC, and BRCA tissues and compared with their corresponding normal tissues.

### 2.11 Molecular docking and visualization

Protein information for the AFCs–cancer prognostic targets was obtained from UniProt,^10^ restricting entries to *Homo sapiens* and “reviewed” (Swiss-Prot) records. Experimentally resolved protein structures were retrieved from the RCSB Protein Data Bank [PDB; (21)].^11^ Structures were selected according to the following criteria: X-ray crystallography, high resolution, near full-length sequence coverage, and one representative structure per target. The selected PDB files were used as macromolecular receptors for docking. The 2D structures of the four AFCs were downloaded from PubChem and converted to PDB format using Open Babel GUI (v3.1.1) (22) to generate ligand files. Receptor structures were prepared in PyMOL (v3.1.3) by removing water molecules, co-crystallized ligands, and other non-receptor heteroatoms, adding hydrogen atoms, and saving the processed structures in PDB format (23). Molecular docking of each AFCs to each target receptor was performed using AutoDock Vina (v1.1.2) (24) with default parameters. Docking poses were ranked by predicted binding affinity; complexes with lower (more negative) binding energies and plausible binding conformations were selected for visualization in PyMOL. Binding energies for the selected complexes were summarized and visualized using the GENESCLOUD platform.^12^ A binding energy ≤−5.0 kcal/mol was used as a threshold to indicate relatively favorable binding.

### 2.12 Cell culture and quantitative real-time PCR (qPCR)

Human lung adenocarcinoma A549 cells were cultured in RPMI-1640 supplemented with 10% fetal bovine serum (FBS) and 1% penicillin/streptomycin. Human colon cancer HCT116 cells and murine Lewis lung carcinoma (LLC) cells were maintained in DMEM containing the same supplements. Human gastric cancer AGS cells were cultured in Ham’s F-12 medium with identical supplements. Human breast cancer MCF-7 cells were maintained in MEM supplemented as above, with the addition of insulin (10 μg/mL). Cell lines were obtained from Wuhan Procell. All cells were maintained at 37 °C in a humidified incubator with 5% CO₂.

For qPCR experiments, A549, HCT116, AGS, and MCF-7 cells were seeded in 6-well plates at 2 × 10⁵ cells/well and cultured for 24 h in complete medium, followed by 24 h in serum-free medium. Cells were treated with 1 μM Allura Red AC (TargetMol, TN1370, China) or 1 μM Tartrazine [TargetMol, TN2258, China; Kwon et al. (59)]. After treatment, cells were washed twice with PBS and total RNA was extracted using VeZol Reagent (Vazyme, R411-01, China). RNA concentration and purity were assessed using a NanoDrop 2000 spectrophotometer (Thermo Fisher Scientific, United States). Reverse transcription was performed using 1 μg of total RNA with the Evo M-MLV RT Kit with gDNA Clean for qPCR (Accurate Biology, AG11705, China). Quantitative PCR was conducted using Hieff® qPCR SYBR Green Master Mix (Yeasen, 11201ES08, China) on a ViiA™ 7 Real-Time PCR System (Thermo Fisher Scientific, United States).

### 2.13 Animal experiment

Eighteen 5-week-old C57BL/6 J mice were purchased from Hunan Silaikejingda Experimental Animal Co., Ltd. and housed at the Laboratory Animal Center of the Second Xiangya Hospital, Central South University. After 1 week of acclimatization, mice were allocated into experimental groups (*n* = 6 per group) and provided with drinking water containing an AFC (Allura Red AC or Tartrazine) at 0.025% (w/v; 0.25 g/L), starting 2 weeks before LLC inoculation and continuing until the end of the experiment (4). Control mice received the same drinking water without AFCs. All drinking water was sterilized by filtration through a 0.22-μm filter. On day 0, LLC cells (5 × 10⁵ cells/mouse) were injected subcutaneously into the axillary region. Tumor volume was measured every 3 days starting on day 7. On day 16, mice were euthanized and tumor tissues were harvested for downstream analyses. All animal procedures were approved by the Animal Ethics Committee of the Second Xiangya Hospital, Central South University (Approval No. 20250352).

## 3 Results

### 3.1 AFCs targets

**Figure 2:**
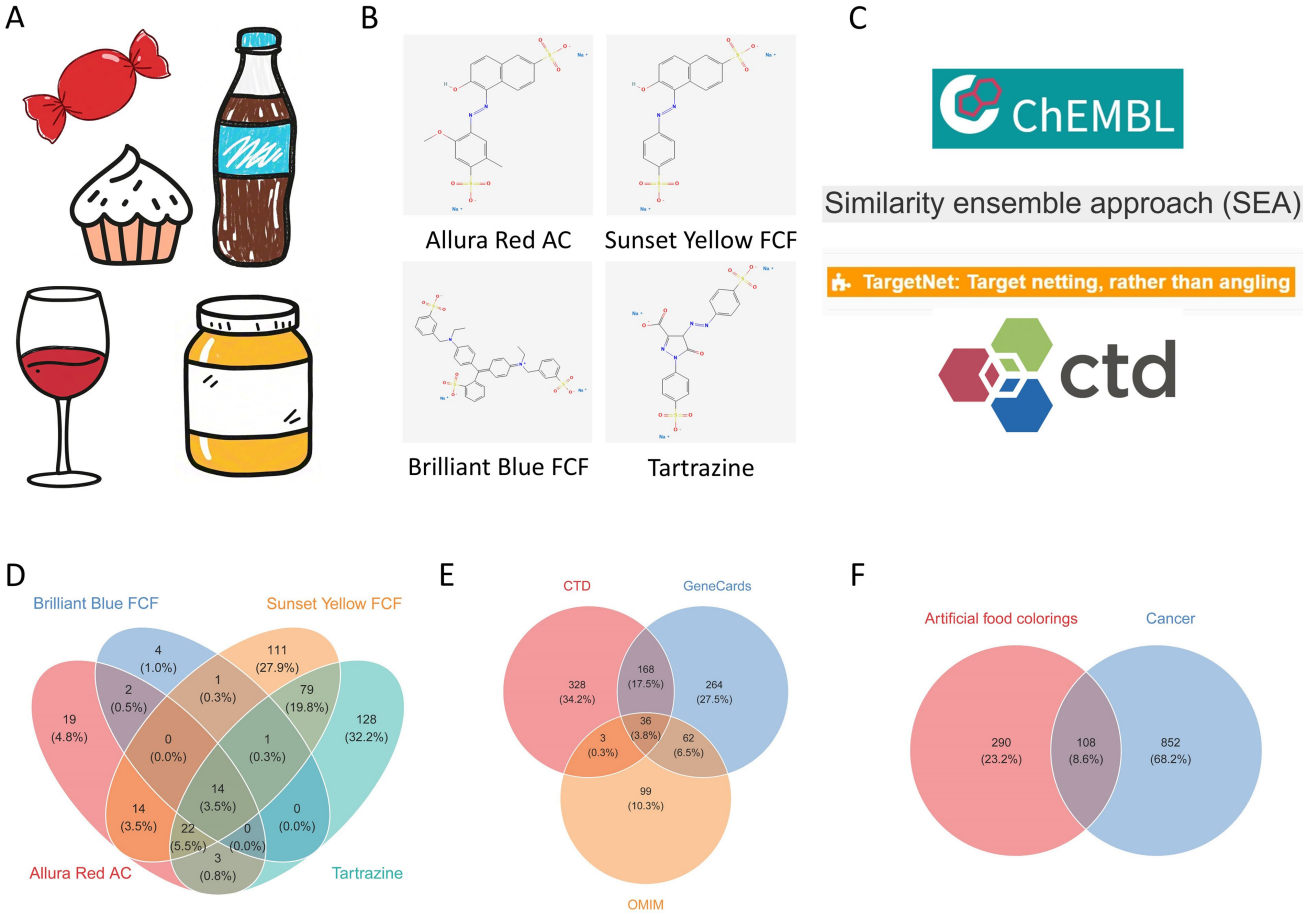
Identification of AFCs-cancer targets. **(A)** Common food products containing AFCs. **(B)** Chemical structures of the four commonly used AFCs. **(C)** Databases used for retrieving AFCs-related targets. **(D)** Venn diagram of targets associated with individual AFCs. **(E)** Venn diagram of cancer-related targets. **(F)** Venn diagram of the overlapping AFCs-cancer targets.

By integrating target information from four databases, a total of 74 targets were identified for Allura Red AC, 242 for Sunset Yellow FCF, 22 for Brilliant Blue FCF, and 249 for Tartrazine. After merging and removing redundancies, 398 unique AFCs-associated targets were obtained (Figures 2A–D).

### 3.2 AFCs-cancer targets

Using “cancer” as the search keyword, 530 targets were retrieved from GeneCards, 200 from OMIM, and 535 from the CTD database. Integration of these datasets resulted in 960 non-redundant cancer-related targets (Figure 2E). Intersection analysis between the 398 AFCs-associated targets and the cancer target set identified 108 shared AFCs–cancer targets (Figure 2F).

### 3.3 Protein–protein interaction (PPI) network

The 108 AFCs–cancer targets were imported into the STRING database to construct a PPI network, with the minimum required interaction score set to the highest confidence level (0.900). After removal of disconnected nodes, the resulting PPI network consisted of 105 nodes (Supplementary Figure 1A). The network was visualized using Cytoscape (v3.8.0), with isolated nodes hidden (Supplementary Figure 1B). Nodes were ranked according to degree centrality, with larger node sizes and darker colors indicating higher levels of connectivity.

### 3.4 GO and KEGG enrichment analysis

**Figure 3:**
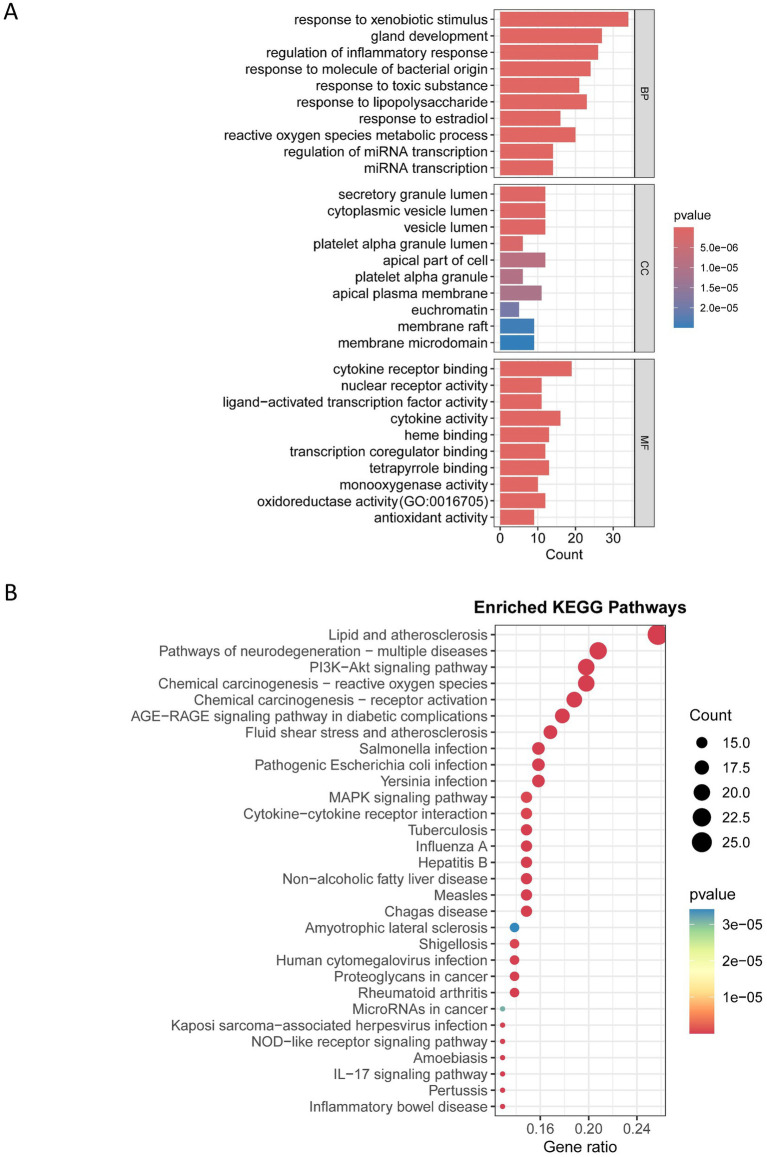
KEGG and GO enrichment analysis of AFCs-cancer targets. **(A)** GO enrichment bar plot. **(B)** KEGG enrichment bubble plot.

GO and KEGG pathway enrichment analyses were conducted for the 108 AFCs–cancer targets and visualized accordingly. Significance was defined as raw *p* < 0.05, consistent with the criteria described in Methods. GO enrichment identified 1,965 significantly enriched biological process (BP) terms, 64 cellular component (CC) terms, and 152 molecular function (MF) terms. The top 10 enriched terms in each GO domain are shown in Figure 3A. Notably, several highly ranked terms were related to xenobiotic response and inflammation, including “response to xenobiotic stimulus,” “regulation of inflammatory response,” “cytokine receptor binding,” and “oxidoreductase activity” (Figure 3A). For KEGG analysis, the top 30 enriched pathways were displayed as a bubble plot (Figure 3B). Enrichment was observed in cancer-relevant signaling pathways, including the “PI3K-Akt signaling pathway,” “MAPK signaling pathway,” “Cytokine–cytokine receptor interaction,” and “IL-17 signaling pathway” (Figure 3B).

### 3.5 Network construction and enrichment analysis of AFCs cancer core targets

**Figure 4:**
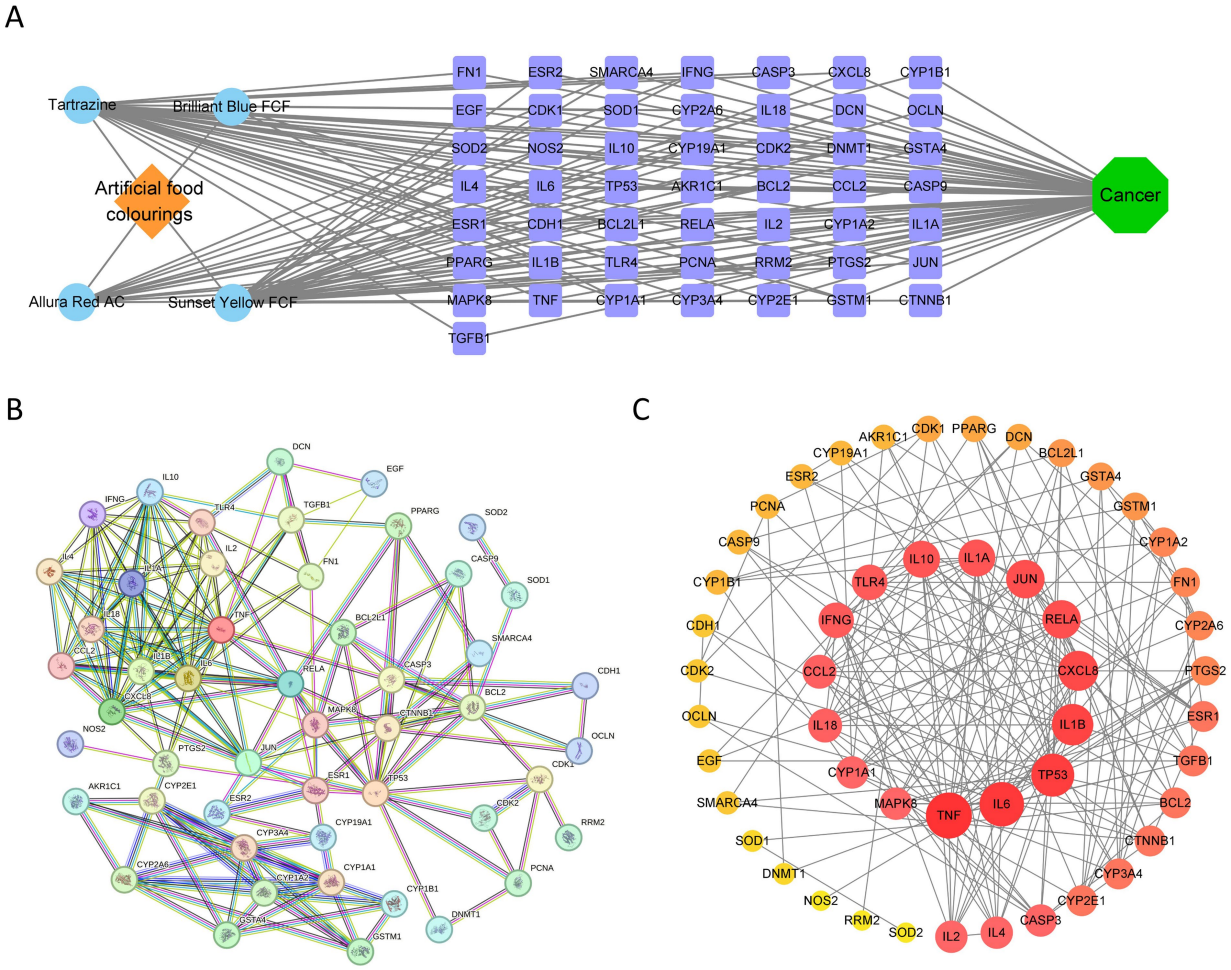
Identification of AFCs-cancer core targets. **(A)** The AFCs-target-cancer network. Orange nodes represent AFCs, blue nodes represent individual AFCs, green nodes represent cancer, and purple nodes represent targets. **(B)** PPI network of AFCs-cancer core targets. **(C)** Visualization of the PPI network for AFCs-cancer core targets using Cytoscape 3.8.0. Nodes with darker colors and larger sizes indicate higher degree values, representing stronger interactions within the network.

**Figure 5:**
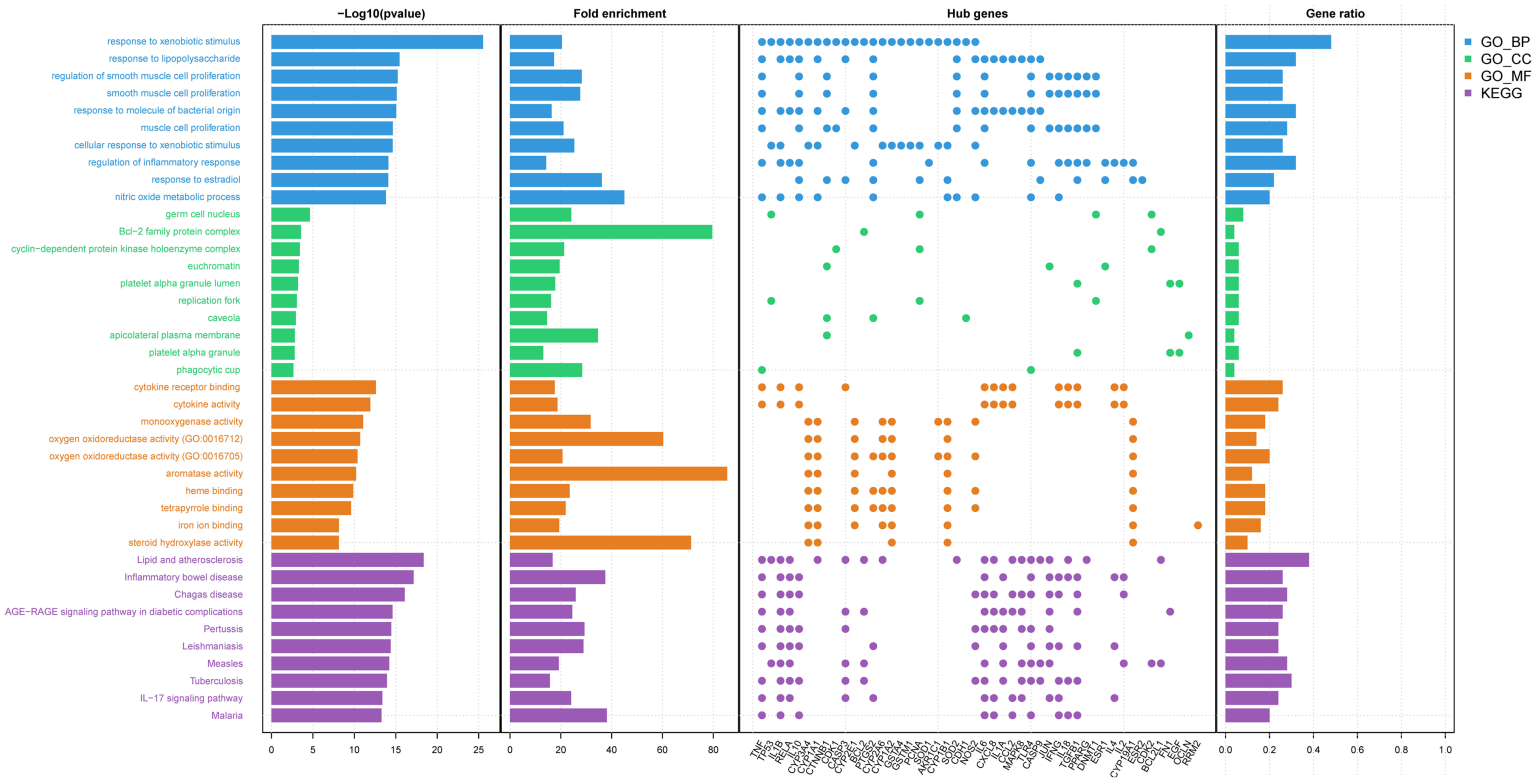
KEGG and GO enrichment analysis of AFCs-cancer core targets.

Topological properties of the AFCs–cancer interaction network were computed in Cytoscape (v3.8.0). The top 50 genes ranked by degree were defined as core AFCs–cancer targets; representative high-degree nodes included TP53, IL6, TNF, JUN, and RELA (Figure 4C). Detailed information and supporting documentation for the AFCs–cancer core targets are provided in Supplementary Table 3. These 50 core targets were used to construct a AFCs–core target–cancer interaction network in Cytoscape (Figure 4A). In parallel, a PPI network was generated in STRING with the interaction confidence threshold set to the highest level (0.900) and visualized in Cytoscape (Figures 4B,C). Functional enrichment analyses were then performed for the 50 core targets. Significance was defined as raw *p* < 0.05, consistent with the enrichment criteria described in Methods. GO enrichment identified 1,918 significantly enriched BP terms, 60 CC terms, and 131 MF terms, and the top 10 terms in each GO domain are presented. Among the most enriched BP terms were “response to xenobiotic stimulus,” “cellular response to xenobiotic stimulus,” and “regulation of inflammatory response.” Enriched CC terms included “Bcl-2 family protein complex” and “cyclin-dependent protein kinase holoenzyme complex,” while enriched MF terms included “cytokine receptor binding,” “cytokine activity,” and “oxygen oxidoreductase activity.” KEGG enrichment identified 138 significantly enriched pathways, and the top 10 pathways are shown in Figure 5.

### 3.6 DEGs of AFCs-cancer core targets

**Figure 6:**
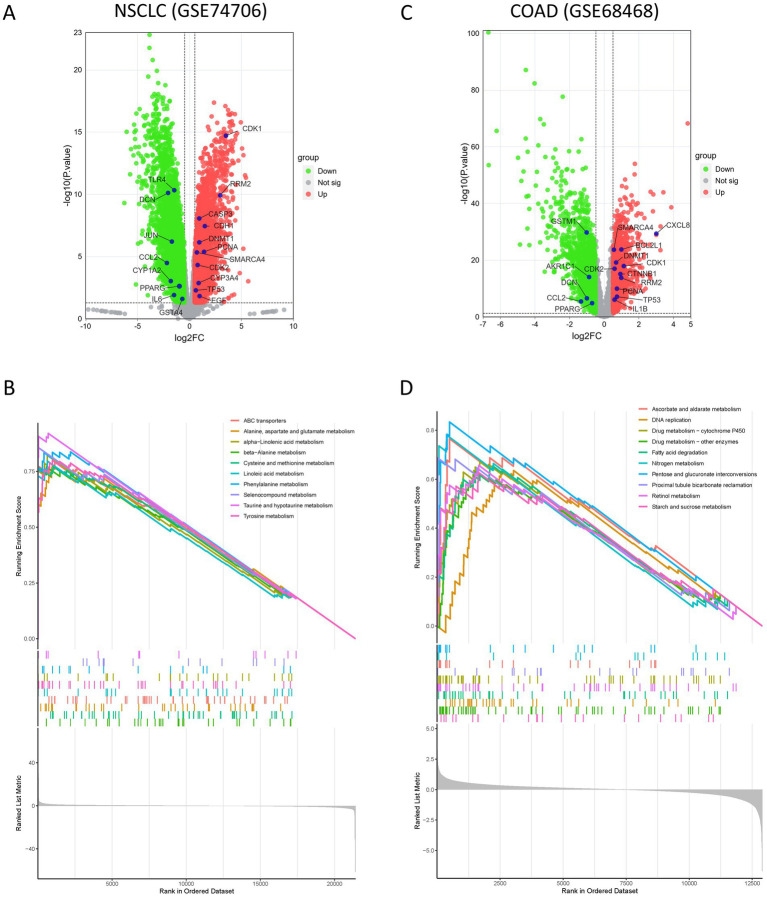
Differential gene expression (DEGs) analysis and GSEA enrichment analysis in the NSCLC dataset (GSE74706) and the COAD dataset (GSE68468). **(A)** Volcano plot of DEGs in the NSCLC dataset. Significantly dysregulated AFCs-Cancer core targets (*p* < 0.05, |Log2FC| > 0.5) are labeled. **(B)** GSEA enrichment plot of the top 10 up-regulated pathways in the NSCLC dataset. **(C)** Volcano plot of DEGs in the COAD dataset. Significantly dysregulated AFCs-Cancer core targets (*p* < 0.05, |Log2FC| > 0.5) are labeled. **(D)** GSEA enrichment plot of the top 10 up-regulated pathways in the COAD dataset.

Differential expression of the AFCs–cancer core targets was evaluated in four cancer types (NSCLC, GC, COAD, and BRCA) using the corresponding GEO datasets (GSE74706, GSE19826, GSE68468, and GSE22820). Volcano plots highlighted distinct expression patterns across cancer types (Figure 6; Supplementary Figure 2). In NSCLC, CDK1, RRM2, CASP3, CDH1, DNMT1, PCNA, SMARCA4, CDK2, CYP3A4, TP53, and EGF were significantly upregulated, whereas TLR4, DCN, JUN, CCL2, CYP1A2, PPARG, IL6, and GSTA4 were significantly downregulated (Figure 6A). In COAD, CXCL8, SMARCA4, BCL2L1, DNMT1, CDK1, CDK2, CTNNB1, RRM2, PCNA, TP53, and IL1B were upregulated, while GSTM1, AKR1C1, DCN, CCL2, and PPARG were downregulated (Figure 6C). In GC, FN1, CTNNB1, DNMT1, IL18, and CYP1B1 were upregulated, whereas OCLN, AKR1C1, GSTA4, and IL1B were downregulated (Supplementary Figure 2A). In BRCA, TGFB1, FN1, CDK1, RRM2, and IL18 were upregulated, while AKR1C1, EGF, PPARG, and PTGS2 were downregulated (Supplementary Figure 2C).

### 3.7 GSEA

GSEA was performed to characterize biological pathways represented by the differential expression profiles across the four cancers. The top 10 significantly upregulated KEGG pathways were selected for visualization (Figure 6; Supplementary Figure 2). In NSCLC, enrichment was observed in pathways including “ABC transporters,” “alpha-linolenic acid metabolism,” “cysteine and methionine metabolism,” and “linoleic acid metabolism” (Figure 6B). In COAD, significantly enriched pathways included “DNA replication,” “drug metabolism—cytochrome P450,” “fatty acid degradation,” and “pentose and glucuronate interconversions” (Figure 6D). In GC, enriched pathways included “beta-alanine metabolism,” “fatty acid metabolism,” “histidine metabolism,” and “propanoate metabolism” (Supplementary Figure 2B). In BRCA, enrichment was observed in “arginine and proline metabolism,” “butanoate metabolism,” “proteasome,” and “tyrosine metabolism” (Supplementary Figure 2D).

### 3.8 Analysis of AS-cancer core targets in common tumors

Expression profiles of the 50 AFCs–cancer core targets across four common cancer types were obtained from GEPIA and visualized as a heatmap using the CNSknowall platform. FN1 exhibited consistently high expression across all four cancers. Additional highly expressed genes included DCN, SOD1, SOD2, CTNNB1, and CDH1. In contrast, the five genes with the lowest expression levels were IL4, CYP1A2, CYP1A1, IL2, and CYP3A4 (Supplementary Figure 3).

### 3.9 Cancer risk prognostic model

**Figure 7:**
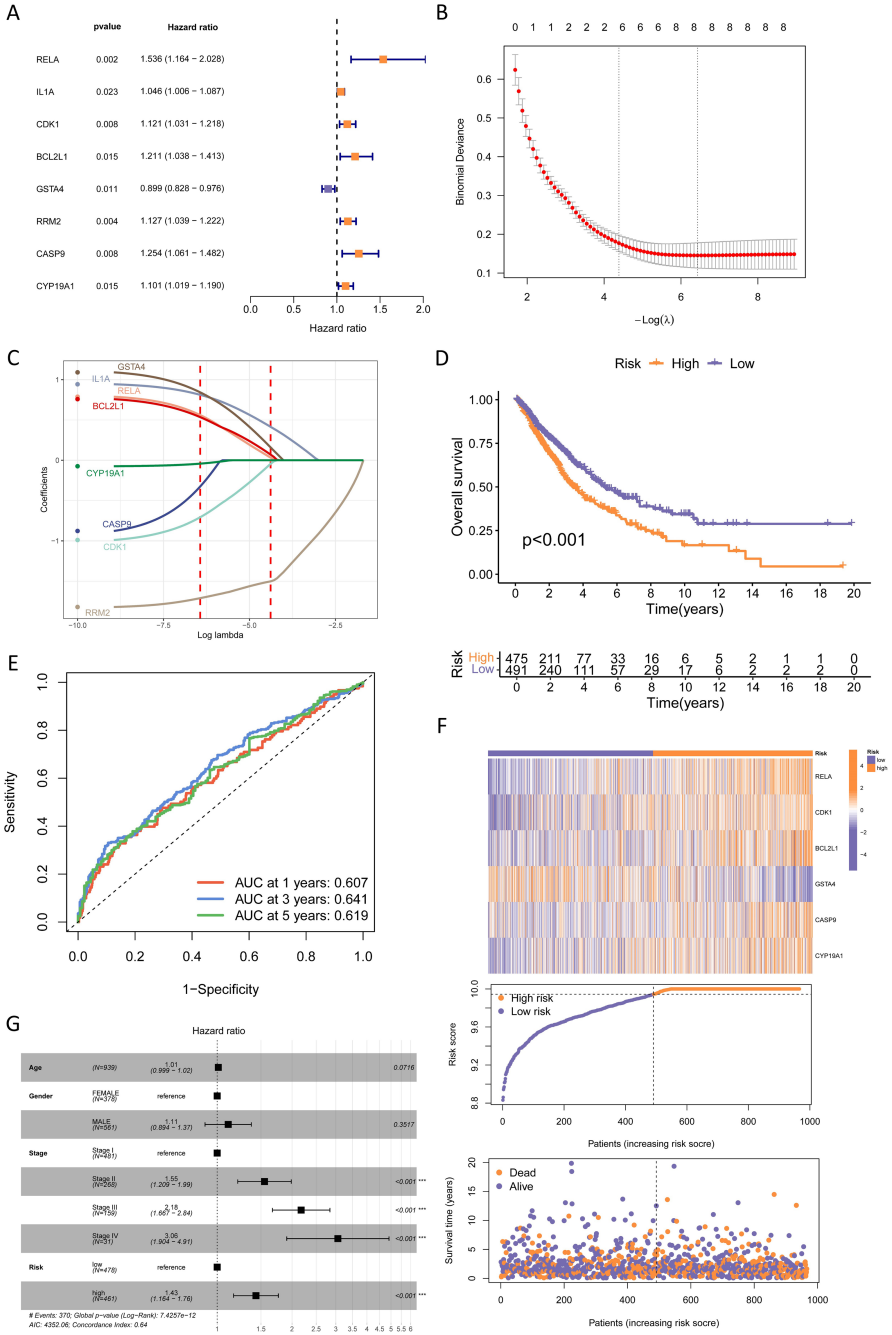
Construction of a prognostic risk model for NSCLC. **(A)** Univariate Cox regression analysis identified eight prognostic genes (*p* < 0.05). **(B)** Coefficient profile plot generated from the LASSO regression analysis, with the optimal lambda (*λ*) value indicated by the right vertical dashed line. **(C)** LASSO regression analysis based on the eight prognostic genes. **(D)** Kaplan–Meier survival analysis comparing high-risk and low-risk groups. **(E)** Time-dependent ROC curves of the prognostic model for 1-, 3-, and 5-year overall survival. **(F)** Visualization of the relationship between patient survival status and risk score, as well as the association between prognostic gene expression and risk score. The dashed line represents the cutoff between low-risk and high-risk groups. **(G)** Multivariate Cox regression analysis of clinical characteristics and risk groups.

**Figure 8:**
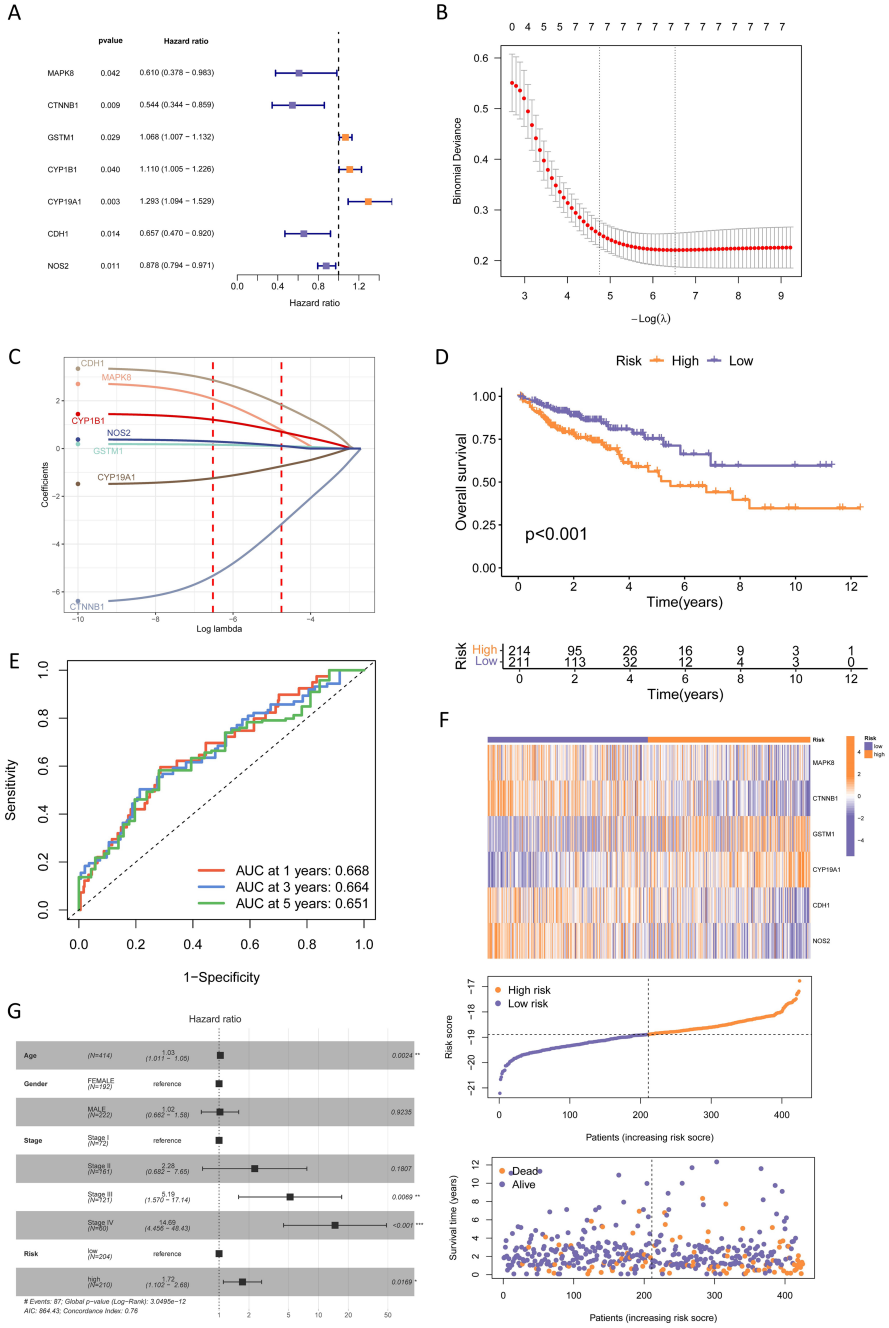
Construction of a prognostic risk model for COAD. **(A)** Univariate Cox regression analysis identified seven prognostic genes (*p* < 0.05). **(B)** Coefficient profile plot generated from the LASSO regression analysis, with the optimal lambda (*λ*) value indicated by the right vertical dashed line. **(C)** LASSO regression analysis based on the 7 prognostic genes. **(D)** Kaplan–Meier survival analysis comparing high-risk and low-risk groups. **(E)** Time-dependent ROC curves of the prognostic model for 1-, 3-, and 5-year overall survival. **(F)** Visualization of the relationship between patient survival status and risk score, as well as the association between prognostic gene expression and risk score. The dashed line represents the cutoff between low-risk and high-risk groups. **(G)** Multivariate Cox regression analysis of clinical characteristics and risk groups.

Univariate Cox regression was performed for the 50 AFCs-associated cancer-related targets (AFCs-cancer core targets) in four TCGA cohorts, and significant OS-associated genes were visualized using forest plots (Figures 7A, 8A; Supplementary Figures 4A, 5A). Prognostic signatures were then established using LASSO regression followed by multivariate Cox modeling, and patients were stratified into high- and low-risk groups based on the median risk score (RS).

In NSCLC, eight genes were significantly associated with OS (*p* < 0.05) (Figure 7A). LASSO regression based on these eight genes is shown in Figures 7B,C. The final multivariate Cox model comprised six genes (RELA, CDK1, BCL2L1, GSTA4, CASP9, and CYP19A1), and the RS was calculated as: RS = 0.409 × RELA + 0.151 × CDK1 + 0.175 × BCL2L1–0.117 × GSTA4 + 0.176 × CASP9 + 0.126 × CYP19A1. Patients in the high-risk group had significantly worse OS than those in the low-risk group (*p* < 0.05) (Figure 7D). Time-dependent ROC analysis yielded AUCs of 0.607 (1-year), 0.641 (3-year), and 0.619 (5-year) (Figure 7E). The risk score distribution indicated that higher expression of RELA, CDK1, BCL2L1, CASP9, and CYP19A1 and lower expression of GSTA4 were associated with increased RS, and higher RS was accompanied by increased mortality (Figure 7F). In multivariate Cox analysis, tumor stage was independent risk factors; the high-risk group showed an HR of 1.43 (95% CI, 1.164–1.76) (Figure 7G).

In COAD, seven genes were significantly associated with OS (*p* < 0.05) (Figure 8A), and LASSO regression results are shown in Figures 8B,C. The final multivariate Cox model included six genes (MAPK8, CTNNB1, GSTM1, CYP19A1, CDH1, and NOS2), with RS defined as: RS = −0.560 × MAPK8–0.560 × CTNNB1 + 0.053 × GSTM1 + 0.185 × CYP19A1–0.350 × CDH1–0.128 × NOS2. High-risk patients had significantly poorer OS (*p* < 0.05) (Figure 8D). The time-ROC AUCs were 0.668 (1-year), 0.664 (3-year), and 0.651 (5-year) (Figure 8E). RS increased with higher expression of CYP19A1 and GSTM1 and lower expression of MAPK8, CTNNB1, CDH1, and NOS2, and higher RS was associated with increased mortality (Figure 8F). Multivariate Cox analysis identified age and tumor stage as independent risk factors, with an HR of 1.72 (95% CI, 1.102–2.68) for the high-risk group (Figure 8G).

In GC, ten genes were significantly associated with OS (*p* < 0.05; Supplementary Figure 4A), and LASSO regression results are provided in Supplementary Figures 4B,C. The final multivariate Cox model comprised four genes (FN1, SMARCA4, CYP19A1, and EGF), and RS was calculated as: RS = 0.106 × FN1–0.327 × SMARCA4 + 0.191 × CYP19A1 + 0.116 × EGF. The high-risk group showed significantly worse OS (*p* < 0.05; Supplementary Figure 4D). Time-ROC analysis yielded AUCs of 0.685 (1-year), 0.642 (3-year), and 0.684 (5-year) (Supplementary Figure 4E). Higher RS corresponded to higher expression of FN1, CYP19A1, and EGF and lower expression of SMARCA4, and was accompanied by increased mortality (Supplementary Figure 4F). In multivariate Cox analysis, age and tumor stage were independent risk factors; the HR for the high-risk group was 1.69 (95% CI, 1.195–2.40; Supplementary Figure 4G).

In BRCA, nine genes were significantly associated with OS (*p* < 0.05) (Supplementary Figure 5A), and LASSO regression results are shown in Supplementary Figures 5B,C. After LASSO regularization, seven genes were retained for multivariate Cox analysis, yielding a final five-gene prognostic signature (IL10, IFNG, IL18, BCL2, and PTGS2). The RS was calculated as: RS = 0.467 × IL10–0.223 × IFNG − 0.167 × IL18–0.168 × BCL2–0.131 × PTGS2. High-risk patients had significantly poorer OS (*p* < 0.05; Supplementary Figure 5D). Time-ROC analysis yielded AUCs of 0.685 (1-year), 0.714 (3-year), and 0.632 (5-year; Supplementary Figure 5E). Higher RS was associated with higher IL10 expression and lower expression of IFNG, IL18, BCL2, and PTGS2, and was accompanied by increased mortality (Supplementary Figure 5F). Multivariate Cox analysis identified age and tumor stage as independent risk factors, and the high-risk group showed an HR of 2.462 (95% CI, 1.7136–3.54) (Supplementary Figure 5G).

Prognostic signatures were derived from cancer-related genes overlapping with AFCs-associated targets that showed significant survival associations. Notably, the identification of these prognostic genes reflects an overlap between AFCs-associated molecular targets and cancer prognostic markers, rather than evidence that AFCs exposure directly confers prognostic relevance.

### 3.10 Importance of prognostic genes interpreted by SHAP value

**Figure 9:**
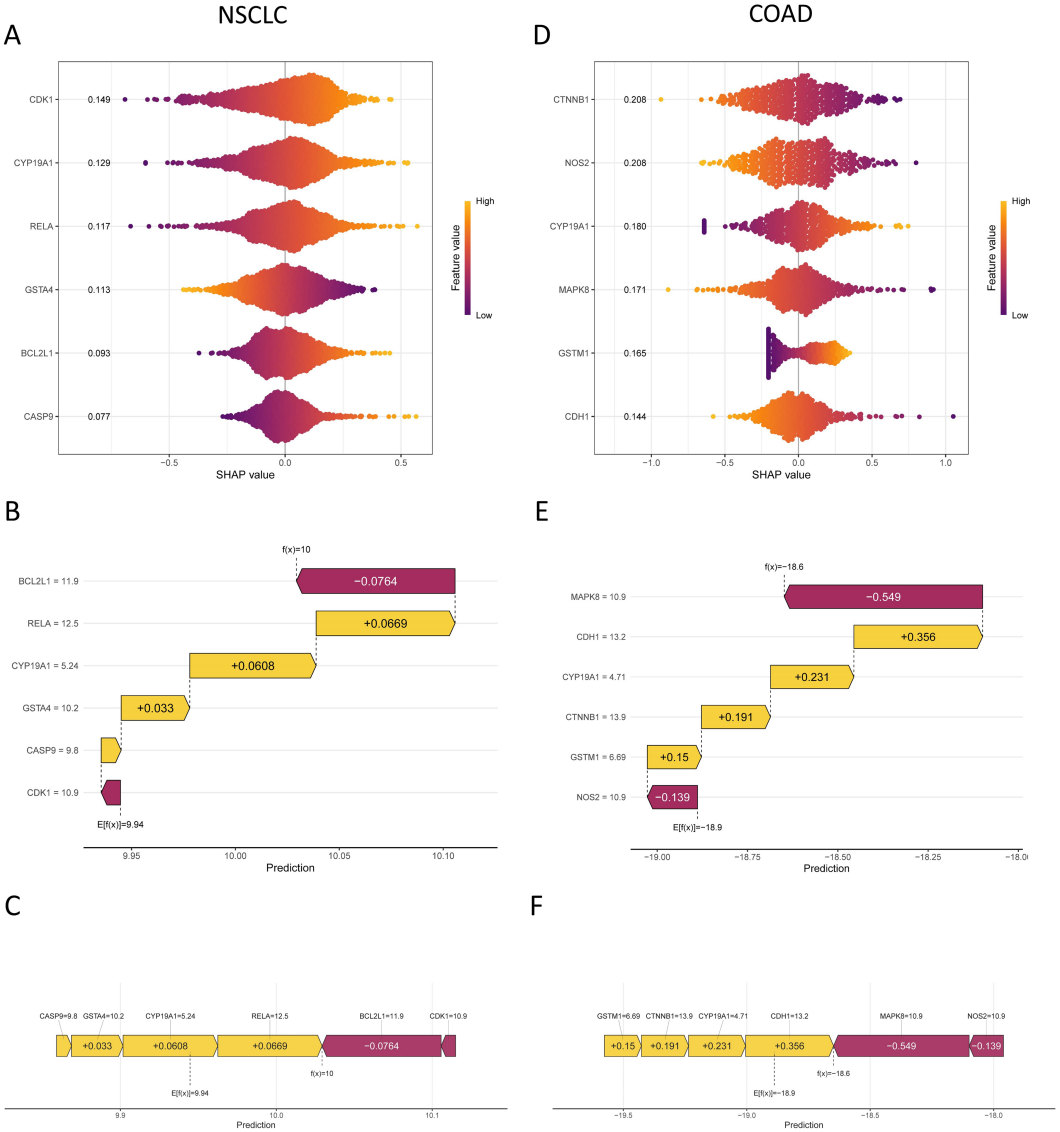
SHAP values of prognostic genes in the prognostic models for NSCLC and COAD. **(A)** SHAP summary plot for the NSCLC model. **(B)** SHAP waterfall plot for the NSCLC model. **(C)** SHAP force plot for the NSCLC model. **(D)** SHAP summary plot for the COAD model. **(E)** SHAP waterfall plot for the COAD model. **(F)** SHAP force plot for the COAD model.

SHAP summary plots were used to quantify the contribution of each prognostic gene to the risk prediction across the four cancer-specific models. In NSCLC, the mean absolute SHAP values were CDK1 (0.149), CYP19A1 (0.129), RELA (0.117), GSTA4 (0.113), BCL2L1 (0.093), and CASP9 (0.077) (Figure 9A). In COAD, the corresponding values were CTNNB1 (0.208), NOS2 (0.208), CYP19A1 (0.180), MAPK8 (0.171), GSTM1 (0.165), and CDH1 (0.144) (Figure 9D). In GC, the values were CYP19A1 (0.241), EGF (0.216), FN1 (0.137), and SMARCA4 (0.134) (Supplementary Figure 6A). In BRCA, the values were IL10 (0.428), IFNG (0.367), BCL2 (0.204), PTGS2 (0.203), and IL18 (0.150) (Supplementary Figure 6D). To further illustrate model interpretability at the individual-sample level, waterfall plots and force plots were generated using the “shapviz” package. These plots depict how each gene contributes to the predicted risk for a given sample: features shown in orange increase the predicted risk, whereas those shown in purple decrease the predicted risk. The sum of feature contributions [i.e., the model output, f(x)] determines the final prediction for each sample (Figures 9B,C and Figures 9E,F; Supplementary Figures 6B,C,E,F).

### 3.11 Protein validation in HPA database

**Figure 10:**
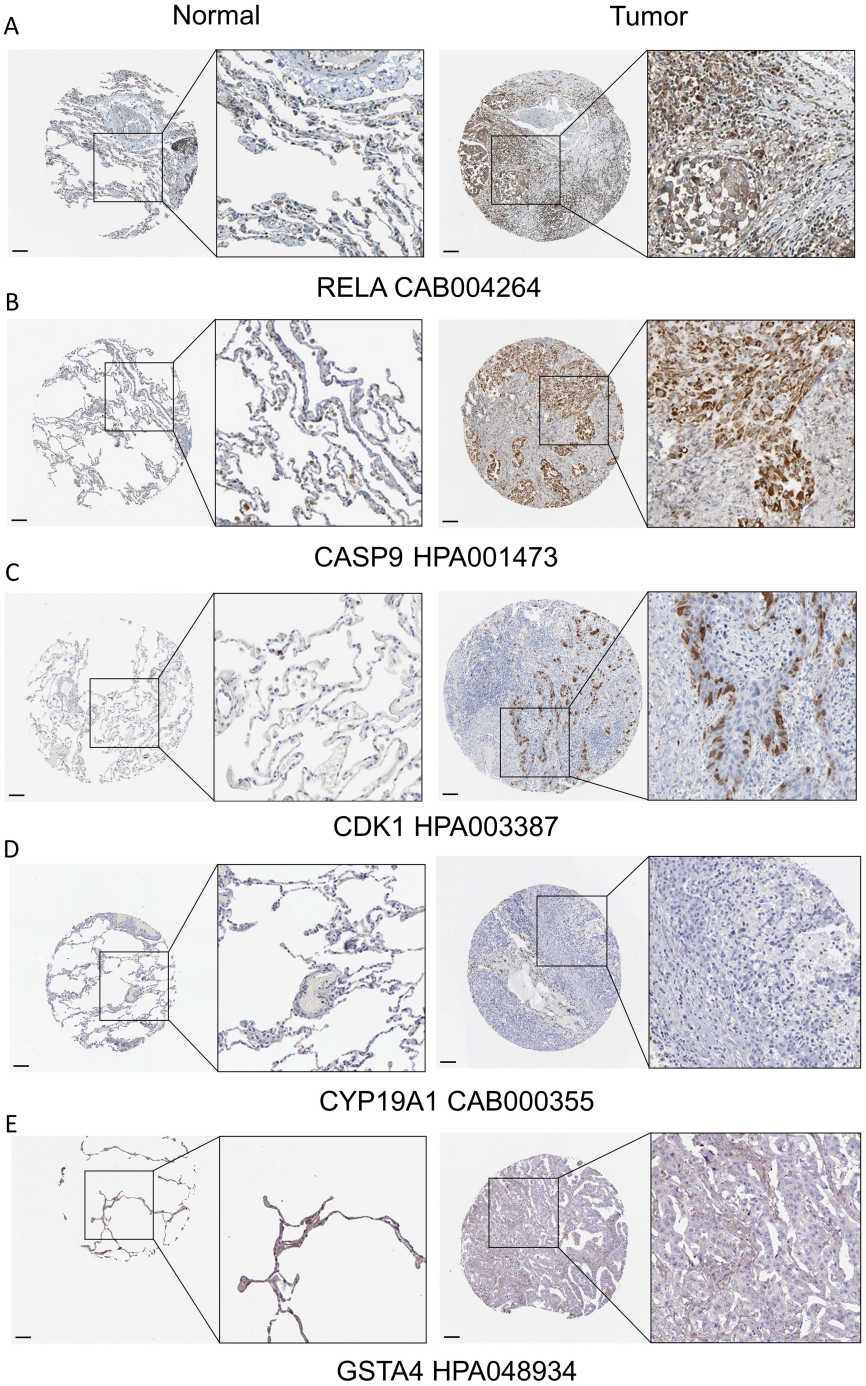
Validation of prognostic gene expression in cancer tissues and matched normal tissues from NSCLC. **(A–E)** Expression levels of RELA, CASP9, CDK1, CYP19A1, and GSTA4 in NSCLC tissues and matched normal lung tissues.

**Figure 11:**
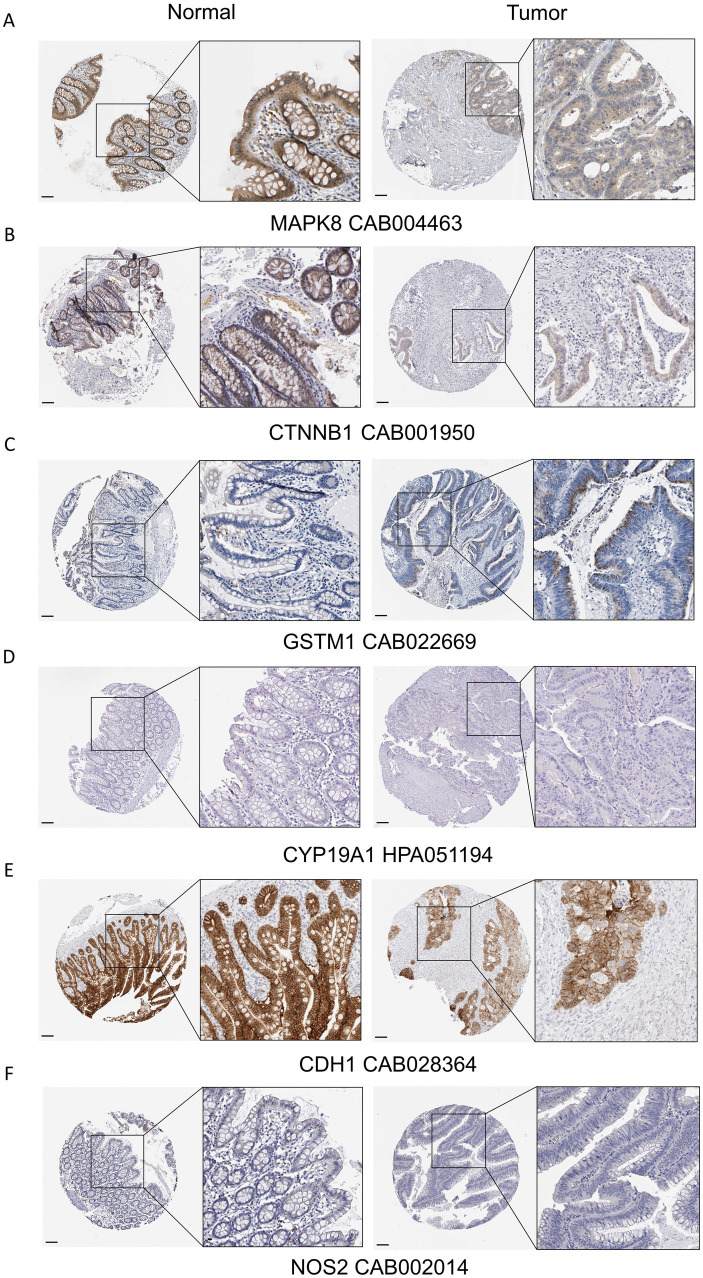
Validation of prognostic gene expression in cancer tissues and matched normal tissues from COAD patients. **(A–F)** Expression levels of MAPK8, CTNNB1, GSTM1, CYP19A1, CDH1, and NOS2 in COAD tissues and matched normal colon tissues.

Immunohistochemistry (IHC) images for selected prognostic genes were retrieved from HPA database. For NSCLC, IHC results for RELA, CASP9, CDK1, CYP19A1, and GSTA4 (antibodies: CAB004264, HPA001473, HPA003387, CAB000355, and HPA048934, respectively) were examined in normal lung and NSCLC tissues. Compared with normal lung tissue, RELA, CASP9, and CDK1 showed higher protein expression in NSCLC tissues (Figures 10A–E). For COAD, IHC data for MAPK8, CTNNB1, GSTM1, CYP19A1, CDH1, and NOS2 (antibodies: CAB004463, CAB001950, CAB022669, HPA051194, CAB028364, and CAB002014) were evaluated in normal colon and COAD tissues. Compared with normal colon tissue, MAPK8, CTNNB1, and CDH1 showed lower expression in COAD tissues, whereas GSTM1 exhibited higher expression (Figures 11A–F). For GC, IHC results for FN1, CYP19A1, and SMARCA4 (antibodies: CAB000126, HPA051194, and CAB004208) were assessed in normal gastric and GC tissues. SMARCA4 showed lower expression in GC tissues, while FN1 showed higher expression relative to normal gastric tissue (Supplementary Figures 7A–C). For BRCA, IHC staining for IL10, IFNG, IL18, BCL2, and PTGS2 (antibodies: CAB013120, CAB010344, HPA003980, CAB000003, and CAB000113) was examined in normal breast and BRCA tissues. Compared with normal breast tissue, IL10 showed higher expression, whereas BCL2 and PTGS2 showed lower expression in BRCA tissues (Supplementary Figures 7D–H). Notably, CYP19A1, GSTA4, NOS2, IFNG, and IL18 did not show clear differential expression between tumor and corresponding normal tissues in the HPA dataset. This may reflect limited available samples or heterogeneity in IHC data, and warrants further investigation. IHC results for EGF were not available in the HPA database and are therefore not presented.

### 3.12 Molecular docking and visualization

**Figure 12:**
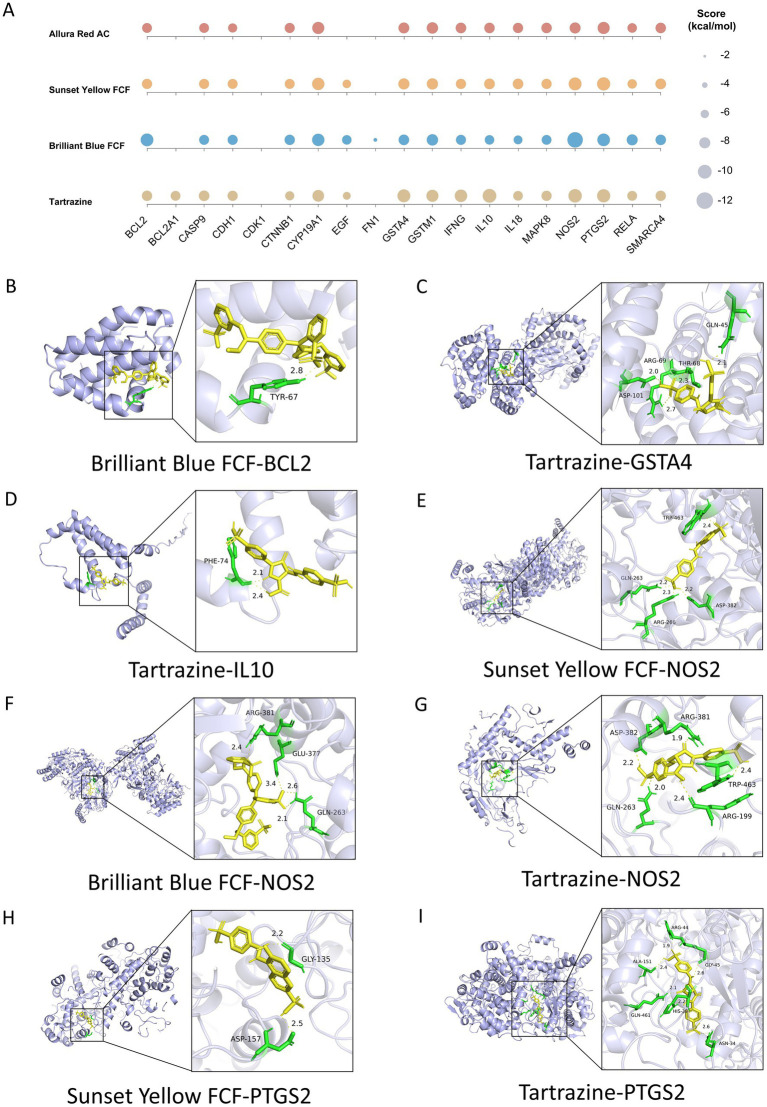
Molecular docking between ACFs and ACF-cancer prognostic targets. **(A)** The multi-axis bubble plot visualizes the binding energies of docked AFCs-cancer related prognostic target complexes, with bubble size corresponding to binding energy magnitude. **(B-I)** 3D schematic diagram of the docked AFCs-cancer related prognostic target complexes.

Molecular docking was performed to further characterize the predicted interactions between 19 prognostic target proteins and four AFCs. Binding energies of all docked complexes are summarized in a multi-axis bubble heatmap (Figure 12A). For detailed visualization, complexes with relatively lower binding energies were selected, including Brilliant Blue FCF–BCL2, Tartrazine–GSTA4, Tartrazine–IL10, Sunset Yellow FCF–NOS2, Brilliant Blue FCF–NOS2, Tartrazine–NOS2, Sunset Yellow FCF–PTGS2, and Tartrazine–PTGS2. Visualization of representative docking poses indicated that AFCs could form hydrogen bonds with specific residues in the target proteins. Brilliant Blue FCF formed one hydrogen bond with TYR67 in BCL2 (2.8 Å) (Figure 12B). Tartrazine interacted with GSTA4 residues ASP101, ARG69, THR68, and GLN45, forming four hydrogen bonds with lengths ranging from 2.0 to 2.7 Å (Figure 12C). Tartrazine formed two hydrogen bonds with PHE74 in IL10 (2.1 and 2.4 Å) (Figure 12D). For NOS2, Sunset Yellow FCF interacted with ARG266, GLN263, TRP463, and ASP382, forming four hydrogen bonds (2.2–2.4 Å) (Figure 12E). Brilliant Blue FCF formed four hydrogen bonds with NOS2 residues ARG381, GLU377, and GLN263 (2.1–3.4 Å) (Figure 12F). Tartrazine bound to NOS2 residues ASP382, ARG381, GLN263, ARG199, and TRP463, forming five hydrogen bonds (1.9–2.4 Å) (Figure 12G). In addition, Sunset Yellow FCF bound to PTGS2 residues ASP157 and GLY135, forming two hydrogen bonds with lengths ranging from 2.2 to 2.5 Å (Figure 12H). Tartrazine interacted with PTGS2 residues ALA151, GLN461, HIS39, ASN34, GLY45, and ARG44, forming six hydrogen bonds (1.9–2.6 Å) (Figure 12I).

### 3.13 External validation

**Figure 13:**
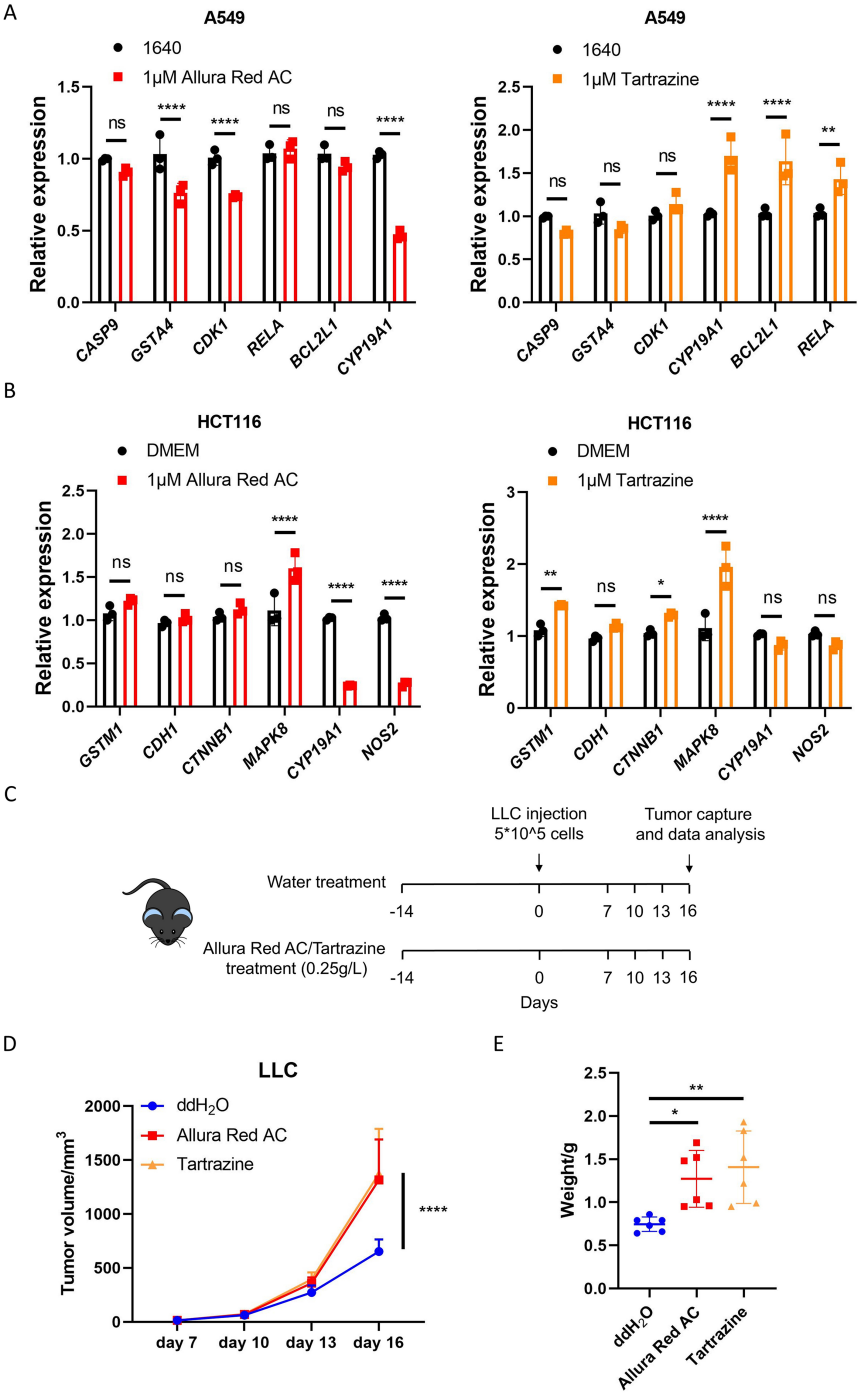
External validation. **(A)** A549 cells stimulated with Allura Red AC and Tartrazine. **(B)** HCT116 cells stimulated with Allura Red AC and Tartrazine. Data are presented as mean ± SD. Statistical significance was determined by two-way ANOVA. *, *p <* 0.05; **, *p <* 0.01; ***, *p <* 0.001; ****, *p <* 0.0001; ns, not significant. **(C)** Animal experimental model diagram. **(D)** Following LLC inoculation in C57BL/6 mice after ACFs treatment. Tumor volume changes were subsequently monitored (*n* = 6 per group). Statistical significance was determined by two-way ANOVA. **(E)** Tumor weight on day 16 after LLC inoculation in C57BL/6 mice after ACFs treatment (*n* = 6 per group). Statistical significance was determined by one-way ANOVA. *, *p* < 0.05; **, *p* < 0.01; ****, *p* < 0.0001.

To externally validate the identified prognostic targets, human lung adenocarcinoma A549 cells, human colon cancer HCT116 cells, human gastric cancer AGS cells, and human breast cancer MCF-7 cells were treated with Allura Red AC and Tartrazine. qPCR analysis showed that exposure to Allura Red AC or Tartrazine modulated the expression of prognostic target genes to varying degrees across the four cell lines, supporting a potential association between AFCs exposure and cancer prognosis-related targets (Figures 13A,B; Supplementary Figures 8A,B).

For *in vivo* validation, LLC cells were used to establish subcutaneous tumor-bearing mouse models (Figure 13C). Tumor-bearing mice receiving drinking water supplemented with Allura Red AC or Tartrazine exhibited faster tumor growth than mice receiving normal drinking water (Figures 13D,E; Supplementary Figure 9). Collectively, these data suggest that high exposure to selected AFCs may be associated with tumor progression in this model. However, these findings should be interpreted cautiously and warrant confirmation in larger, rigorously designed experiments.

## 4 Discussion

AFCs are widely used in food processing, including canned fruits, jams, confectionery, baked goods, beverages, and ice cream. Despite regulatory limits on permitted types and usage levels in jurisdictions such as the United States, Europe, and China, ongoing debate remains regarding the potential health risks associated with widespread AFCs production and consumption (2). Reported concerns include associations with childhood hyperactivity and cancer-related outcomes (2). Notably, several synthetic colorants have been restricted or banned over time because of chronic toxicity or safety concerns. Sudan dyes, a class of industrial colorants, were reported as contaminants in multiple food products in the early 2000s, despite having been prohibited for use in food in the European Union since 1995 (25). Experimental studies have shown that Sudan I can induce malignant tumors in the liver and bladder of rodents, and it has been classified as Group 3 by the International Agency for Research on Cancer (IARC) (25, 26). Continued reports of sporadic illegal use underscore the challenges of enforcement and the need for sustained regulatory oversight (27, 28). Citrus Red No. 2 was permitted by FDA for coloring orange peels under strict use limitations, whereas it is prohibited in China and Europe, consistent with its IARC Group 2B classification and evidence of tumor induction in animal studies (29). Erythrosine, another commonly used AFCs in candies, pastries, and beverages, has been associated with neurobehavioral outcomes in children in prior studies (16, 20). Given ongoing safety considerations, regulatory policies regarding certain synthetic colorants have continued to evolve across regions. The FDA plans to phase out several widely used artificial colorants by 2026, including the four high-exposure ACFs examined in this study, to promote a shift toward more natural and safer food ingredients (8). In Europe and China, these four high-exposure ACFs are not yet prohibited; however, European regulations require products containing them to carry warning labels (Regulation No 1333/2008, 2008; (9)). Against this background, the present study integrates multi-omics analyses, network-based approaches, and TCGA-derived prognostic assessment, together with bioinformatic prediction and *in vitro*/in vivo validation, to investigate potential links between high-exposure AFCs and common cancers. These findings may contribute evidence relevant to future safety evaluations and regulatory decision-making.

To the best of our knowledge, this study represents the first systematic investigation of the potential association between AFCs and cancer risk through an integrative, multi-level analytical framework. By consolidating target information from multiple databases (CTD, ChEMBL, SEA, and TargetNet), we identified 398 AFCs-related targets and 960 cancer-related targets, with 108 shared AFCs–cancer targets. Subsequent network-based screening further refined these to 50 core targets. Functional enrichment analyses revealed that these targets were significantly involved in cancer-associated biological processes and signaling pathways, including xenobiotic response, cell cycle regulation, and key oncogenic pathways such as PI3K–Akt and MAPK signaling. Focusing on four common cancers with high global incidence and mortality (NSCLC, COAD, GC, and BRCA), we further demonstrated that multiple core targets were aberrantly expressed in tumor tissues and that their expression profiles were significantly associated with unfavorable clinical outcomes based on TCGA survival data. Collectively, these findings suggest that high-exposure AFCs may be linked to cancer-related molecular networks and adverse prognosis, providing new insights into potential biological mechanisms underlying AFCs-associated cancer risk.

Among the enriched GO terms, the “cyclin-dependent protein kinase holoenzyme complex” was particularly notable. Cyclin-dependent kinases (CDKs) are central regulators of cell cycle progression and transcriptional control and are broadly categorized into cell cycle–related CDKs (e.g., CDK1, CDK2, CDK4, and CDK6) and transcription-associated CDKs (30, 31). Cell cycle–related CDKs drive orderly transitions through the different phases of the cell cycle by forming complexes with specific cyclins, which confer substrate specificity and temporal regulation (30, 32). Dysregulation of CDK–cyclin activity is a well-established hallmark of cancer, contributing to uncontrolled proliferation and genomic instability. Aberrant activation of CDK4 has been reported in glioblastoma and melanoma (33), while cyclin D1, a key binding partner of CDK4 and CDK6, is frequently overexpressed in breast cancer, head and neck squamous cell carcinoma, and esophageal cancer (31, 33). Importantly, the clinical success of CDK4/6 inhibitors such as ribociclib in hormone receptor–positive, HER2-negative advanced breast cancer underscores the translational relevance of this pathway (34). The enrichment of AFCs–cancer core targets within CDK-related complexes therefore suggests that AFCs-associated molecular perturbations may contribute to tumorigenesis by interfering with cell cycle control and proliferative signaling.

KEGG pathway analysis further highlighted significant enrichment of the PI3K–Akt signaling pathway, a central regulator of multiple cancer hallmarks (35). Aberrant activation of PI3K–Akt signaling promotes tumor initiation and progression through diverse mechanisms, including suppression of FOXO1 nuclear translocation and transcriptional activity, leading to deregulated expression of downstream targets involved in proliferation and survival (36, 37). Previous studies have demonstrated that oncogenic factors such as KDM5A can drive hepatocellular carcinogenesis by modulating the miR-433–FXYD3–PI3K–Akt axis (38). In addition, Akt-mediated phosphorylation of CHK1 attenuates cell cycle checkpoint control, thereby facilitating tumor cell proliferation (39), while activation of downstream effectors such as GSK3, TSC2, and MDM2 further amplifies proliferative and survival signals (40–42). Beyond cell proliferation, PI3K–Akt signaling contributes to metabolic reprogramming by upregulating nutrient transporters and metabolic enzymes, enabling cancer cells to meet the anabolic demands of rapid growth (43). This pathway is also critically involved in angiogenesis, metastasis, therapeutic resistance, immune evasion, and shaping of the inflammatory tumor microenvironment (44–46). The enrichment of PI3K–Akt signaling among AFCs–cancer core targets therefore provides a plausible mechanistic link between AFCs exposure and cancer-related biological processes.

Furthermore, we integrated transcriptomic and clinical outcome data from TCGA and constructed cancer-specific prognostic models using a standard workflow comprising univariate Cox regression, LASSO regularization, and multivariate Cox proportional hazards regression. Across NSCLC, COAD, GC, and BRCA, the resulting risk signatures consistently stratified patients into high- and low-risk groups with significantly different overall survival, supporting a robust association between AFCs–cancer core targets and clinical prognosis. In NSCLC, the final prognostic model consisted of RELA, CDK1, BCL2L1, GSTA4, CASP9, and CYP19A1. Several components of this signature have well-established links to lung cancer biology. RELA, a key subunit of NF-κB, has been associated with unfavorable prognosis in NSCLC (47). CDK1 is a central regulator of cell-cycle progression and has been implicated as a therapeutic vulnerability in lung cancer (48). BCL2L1 (BCL-xL), an anti-apoptotic member of the BCL2 family, promotes tumor cell survival, and its inhibition can induce apoptosis in lung cancer cells (49, 50). CASP9 isoforms have also been reported to influence tumor-promoting signaling; for instance, caspase 9b can activate NF-κB and cooperate with oncogenic KRAS to facilitate lung tumorigenesis (51). In addition, polymorphisms in CYP19A1 have been associated with increased lung cancer susceptibility in the Chinese Han population (52, 53). Consistent with these reports, higher risk scores in our NSCLC model corresponded to higher expression of RELA, CDK1, BCL2L1, CASP9, and CYP19A1 and lower expression of GSTA4, collectively supporting the biological plausibility of the signature and its relevance to adverse NSCLC outcomes. In COAD, the final model incorporated MAPK8, CTNNB1, GSTM1, CYP19A1, CDH1, and NOS2. MAPK8 is a risk factor for melanosis coli (54). CTNNB1 alterations are frequent in colorectal cancer and can drive constitutive Wnt signaling, which has been linked to aggressive phenotypes and poor prognosis (55, 56). GSTM1 deficiency has been reported to increase colorectal cancer risk (57). Moreover, CYP19A1-mediated estrogen biosynthesis may promote immune evasion and tumor progression through upregulation of immunoregulatory factors via the GPR30–AKT axis (58). In our model, increased risk scores were associated with higher CYP19A1 and GSTM1 expression and lower MAPK8, CTNNB1, CDH1, and NOS2 expression, further supporting that AFCs–cancer core targets capture clinically relevant biology in COAD. Similar prognostic associations were observed in GC and BRCA, indicating that AFCs–cancer core targets may contribute to unfavorable outcomes across multiple tumor contexts. It is important to emphasize that the identified prognostic genes reflect an intersection between ACF-associated targets and established cancer prognostic markers, rather than direct evidence that ACFs exposure causally affects patient outcomes via these genes.

To provide additional support beyond transcriptomic associations, we examined protein expression patterns of key prognostic genes using the Human Protein Atlas and experimentally assessed gene expression changes by qPCR following exposure to Allura Red AC or Tartrazine in cancer cell lines. These results suggested partial concordance between mRNA- and protein-level patterns and indicated that selected AFCs could modulate prognosis-related targets *in vitro*. Furthermore, in a subcutaneous LLC tumor model, exposure to Allura Red AC or Tartrazine via drinking water was associated with accelerated tumor growth compared with controls. Notably, these *in vivo* findings should be interpreted cautiously, as they represent preliminary evidence; definitive conclusions will require larger cohorts and long-term exposure designs to more rigorously evaluate causality and dose–response relationships.

This study presents an integrative framework to investigate potential links between commonly used AFCs and cancer-related molecular networks by combining multi-database mining, machine-learning–assisted analyses, network toxicology, TCGA-based prognostic assessment, and HPA validation. Across four common cancers (NSCLC, COAD, BRCA, and GC), we identified shared AFCs–cancer targets and highlighted cancer-relevant functional themes, including cell-cycle regulation (e.g., the cyclin-dependent protein kinase holoenzyme complex) and oncogenic signaling pathways (e.g., PI3K–Akt pathway). Collectively, these findings suggest that high-exposure AFCs may be associated with cancer-related biological processes and adverse clinical outcomes, providing evidence that may inform future safety evaluations and regulatory decision-making for AFCs.

Several limitations should be acknowledged. First, although multiple bioinformatic strategies were applied to characterize associations between AFCs-related targets and cancer outcomes, the predominantly computational nature of the study does not establish causality. Second, TCGA clinical and prognostic data are not fully representative of global populations, which may limit the generalizability of our findings across diverse ancestries. Third, while we focused on four high-exposure AFCs (Allura Red AC, Brilliant Blue FCF, Tartrazine, and Sunset Yellow FCF), potential heterogeneity related to different AFCs types, exposure levels, and dose–response relationships were not comprehensively evaluated. Fourth, due to sample size constraints in certain datasets, we did not conduct detailed subgroup analyses by molecular subtype or other clinically relevant strata. Finally, given that the principal findings are largely associative, further validation in well-controlled experimental systems, including rigorous cellular experiments and large-sample, long-term exposure animal studies, is required to substantiate the proposed mechanisms and clarify the nature of the relationship between AFCs exposure and cancer risk.

Based on the findings of this study, it may be prudent for consumers, particularly children and other potentially vulnerable groups, to limit excessive intake of foods containing AFCs, given ongoing uncertainties regarding their long-term health effects. From an industry perspective, manufacturers should consider reducing the use of AFCs where feasible and prioritizing the adoption of safer alternatives, including natural colorants, while ensuring product quality and compliance with applicable standards. At the regulatory level, our results support the need for continued re-evaluation of the safety profiles of widely used AFCs, strengthened risk assessment and surveillance frameworks, and evidence-based policies that encourage a gradual transition toward safer coloring strategies. Public health efforts that improve transparency and consumer awareness (e.g., clearer labeling and education initiatives) may also help reduce unnecessary exposure.

## 5 Conclusion

In this study, we implemented an integrative strategy combining multi-database mining, machine-learning–assisted analyses, network toxicology, TCGA-based prognostic assessment, and external validation to investigate potential links between commonly used AFCs and cancer. Our results identify shared AFCs–cancer targets and cancer-relevant pathways, suggesting that high-exposure AFCs may be associated with biological processes related to tumor development and unfavorable prognosis in NSCLC, COAD, BRCA, and GC. These findings provide evidence that may inform future safety evaluation and regulatory decision-making regarding AFC use.

## Data Availability

The original contributions presented in the study are included in the article/Supplementary material, further inquiries can be directed to the corresponding author.

## Footnotes

1
https://ctdbase.org/

2
https://pubchem.ncbi.nlm.nih.gov/

3
https://www.ebi.ac.uk/chembl/

4
https://sea.bkslab.org/

5
http://targetnet.scbdd.com/

6
https://www.genecards.org/

7
https://www.omim.org/

8
https://www.cnsknowall.com/#/HomePage

9
https://www.proteinatlas.org/

10
https://www.uniprot.org/

11
https://www.rcsb.org/

12
https://www.genescloud.cn

## Supplementary material

SUPPLEMENTARY FIGURE 1 Identification of AFCs-cancer targets. **(A)** PPI network of AFCs-cancer targets. **(B)** Visualization of the PPI network for AFCs-cancer targets using Cytoscape 3.8.0. Nodes with darker colors and larger sizes indicate higher degree values, representing stronger interactions within the network.

SUPPLEMENTARY FIGURE 2 Differential gene expression (DEGs) analysis and GSEA enrichment analysis in the GC dataset (GSE19826) and the BRCA dataset (GSE22820). **(A)** Volcano plot of DEGs in the GC dataset. Significantly dysregulated AFCs-Cancer core targets (*p* < 0.05, |Log2FC| > 0.5) are labeled. **(B)** GSEA enrichment plot of the top 10 up-regulated pathways in the GC dataset. **(C)** Volcano plot of DEGs in the BRCA dataset. Significantly dysregulated AFCs-Cancer core targets (*p* < 0.05, |Log2FC| > 0.5) are labeled. **(D)** GSEA enrichment plot of the top 10 up-regulated pathways in the BRCA dataset.

SUPPLEMENTARY FIGURE 3 Heatmap visualization of differential expression patterns for the 50 AFCs-Cancer core targets in five common cancers (LUSC, LUAD, COAD, BRCA, STAD).

SUPPLEMENTARY FIGURE 4 Construction of a prognostic risk model for GC. **(A)** Univariate Cox regression analysis identified 10 prognostic genes (*p* < 0.05). **(B)** Coefficient profile plot generated from the Lasso regression analysis, with the optimal lambda (*λ*) value indicated by the right vertical dashed line. **(C)** Lasso regression analysis based on the 10 prognostic genes. **(D)** Kaplan–Meier survival analysis comparing high-risk and low-risk groups. **(E)** Time-dependent ROC curves of the prognostic model for 1-, 3-, and 5-year overall survival. **(F)** Visualization of the relationship between patient survival status and risk score, as well as the association between prognostic gene expression and risk score. The dashed line represents the cutoff between low-risk and high-risk groups. **(G)** Multivariate Cox regression analysis of clinical characteristics and risk groups.

SUPPLEMENTARY FIGURE 5 Construction of a prognostic risk model for BRCA. **(A)** Univariate Cox regression analysis identified nine prognostic genes (*p* < 0.05). **(B)** Coefficient profile plot generated from the Lasso regression analysis, with the optimal lambda (*λ*) value indicated by the right vertical dashed line. **(C)** Lasso regression analysis based on the seven prognostic genes. **(D)** Kaplan–Meier survival analysis comparing high-risk and low-risk groups. **(E)** Time-dependent ROC curves of the prognostic model for 1-, 3-, and 5-year overall survival. **(F)** Visualization of the relationship between patient survival status and risk score, as well as the association between prognostic gene expression and risk score. The dashed line represents the cutoff between low-risk and high-risk groups. **(G)** Multivariate Cox regression analysis of clinical characteristics and risk groups.

SUPPLEMENTARY FIGURE 6 SHAP values of prognostic genes in the prognostic models for gastric cancer and breast cancer. **(A)** SHAP summary plot for the gastric cancer model. **(B)** SHAP waterfall plot for the gastric cancer model. **(C)** SHAP force plot for the gastric cancer model. **(D)** SHAP summary plot for the breast cancer model. **(E)** SHAP waterfall plot for the breast cancer model. **(F)** SHAP force plot for the breast cancer model.

SUPPLEMENTARY FIGURE 7 Validation of prognostic gene expression in cancer tissues and matched normal tissues from GC and BRCA. **(A–C)** Expression levels of FN1, CYP19A1, and SMARCA4 in gastric cancer tissues and matched normal gastric tissues. **(D–H)** Expression levels of IL10, IFNG, IL18, BCL2, and PTGS2 in breast cancer tissues and matched normal breast tissues.

SUPPLEMENTARY FIGURE 8 qPCR validation of the effects of AFCs on the expression of AFCs-cancer prognostic targets in AGS cells and MCF-7 cells. Data are presented as mean ± SD. Statistical significance was determined by two-way ANOVA. *, *p* < 0.05; **, *p* < 0.01; ****, *p* < 0.0001; ns, not significant.

SUPPLEMENTARY FIGURE 9 Representative images of tumors collected on day 16 after LLC inoculation in C57BL/6 mice treated with AFCs (*n* = 6 per group).
