# Supplementary figures and images for: A multi-omics approach elucidates the link between artificial food colorings and common cancers
Source: Front Nutr. 2026 Feb 5;13:1743416. doi: 10.3389/fnut.2026.1743416 (PMC12916573; doi:10.3389/fnut.2026.1743416)

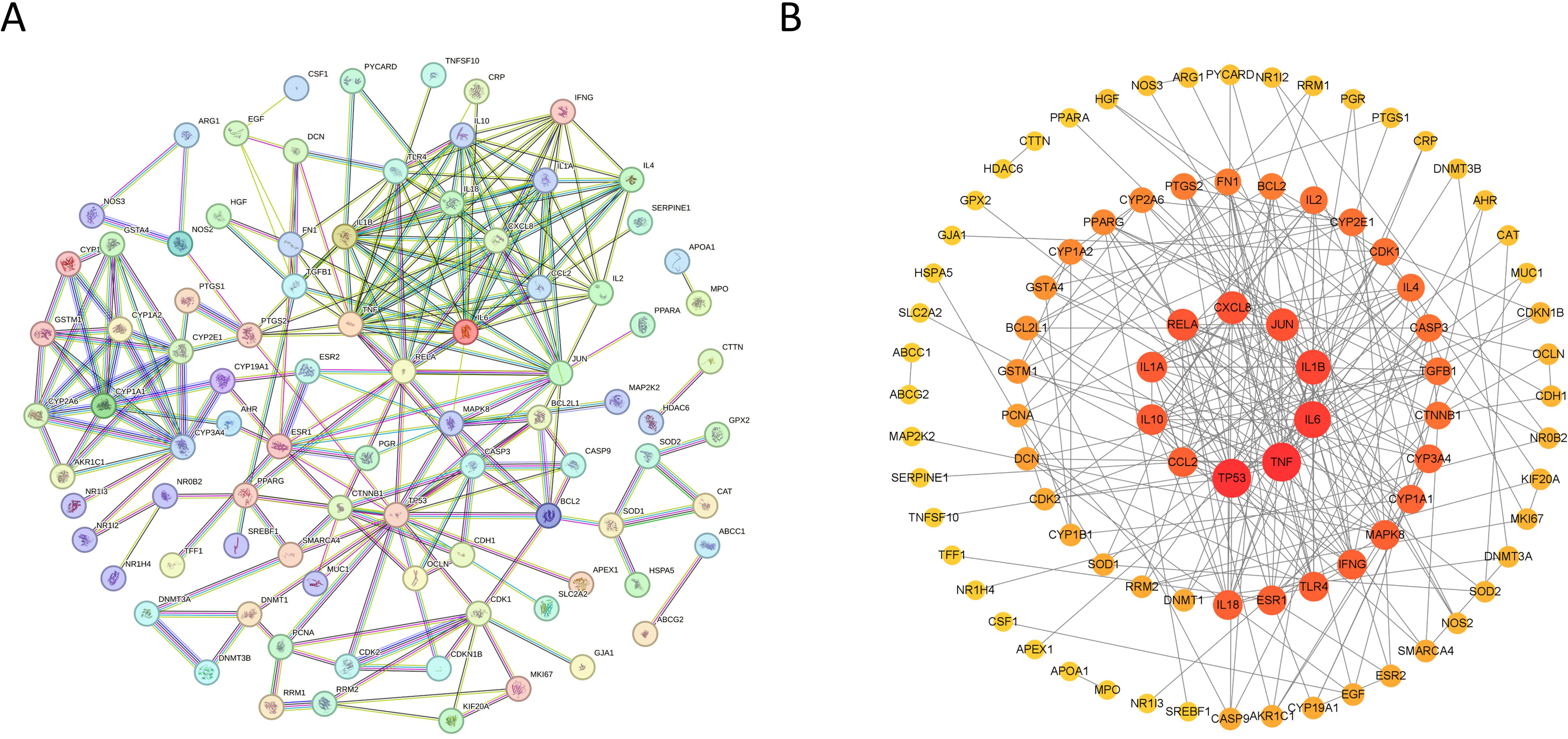

Supplement: SUPPLEMENTARY FIGURE 1 — Identification of AFCs-cancer targets. (A) PPI network of AFCs-cancer targets. (B) Visualization of the PPI network for AFCs-cancer targets using Cytoscape 3.8.0. Nodes with darker colors and larger sizes indicate higher degree values, representing stronger interactions within the network. [file Image_1.JPEG]

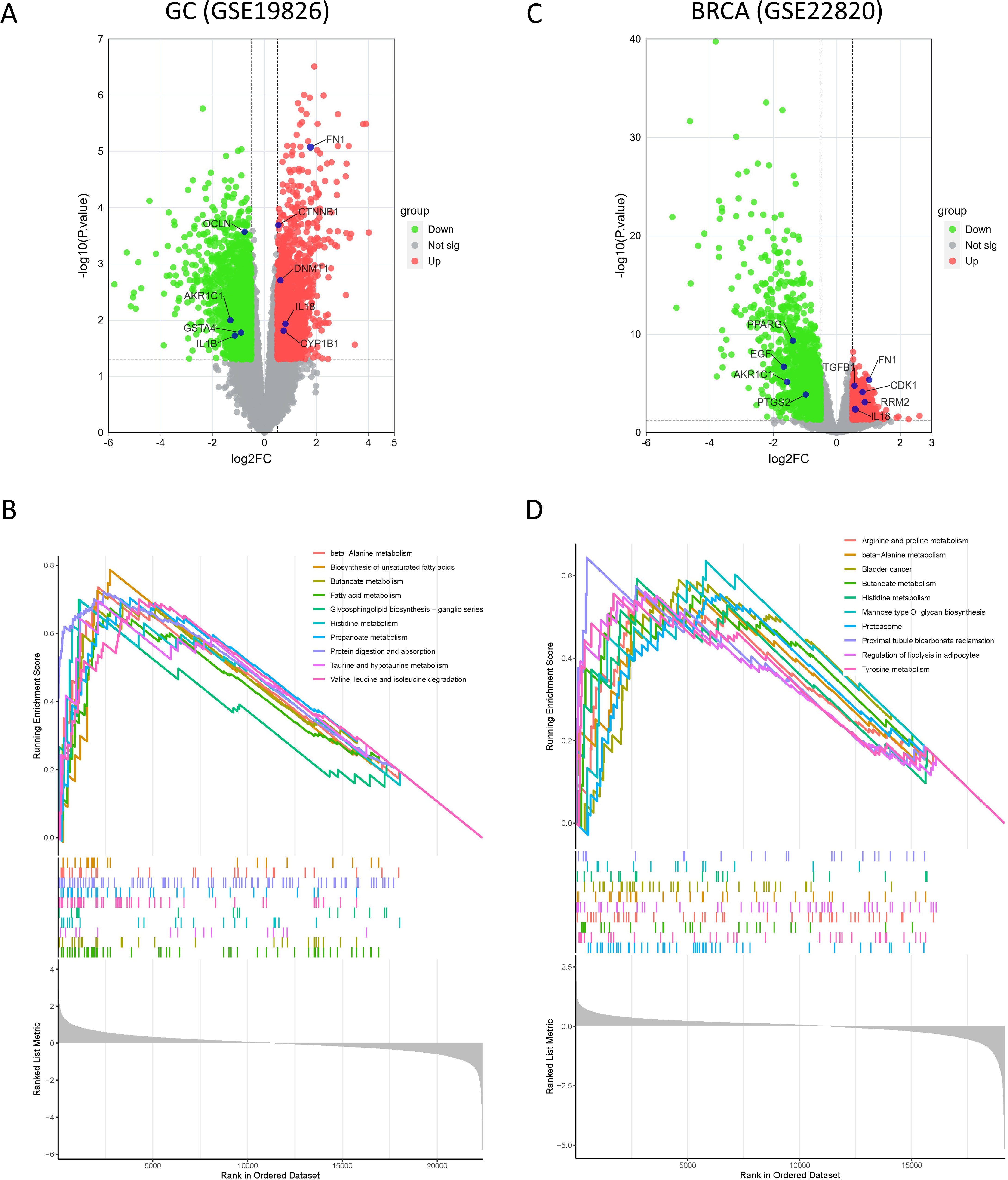

Supplement: SUPPLEMENTARY FIGURE 2 — Differential gene expression (DEGs) analysis and GSEA enrichment analysis in the GC dataset (GSE19826) and the BRCA dataset (GSE22820). (A) Volcano plot of DEGs in the GC dataset. Significantly dysregulated AFCs-Cancer core targets (p < 0.05, |Log2FC| > 0.5) are labeled. (B) GSEA enrichment plot of the top 10 up-regulated pathways in the GC dataset. (C) Volcano plot of DEGs in the BRCA dataset. Significantly dysregulated AFCs-Cancer core targets (p < 0.05, |Log2FC| > 0.5) are labeled. (D) GSEA enrichment plot of the top 10 up-regulated pathways in the BRCA dataset. [file Image_2.JPEG]

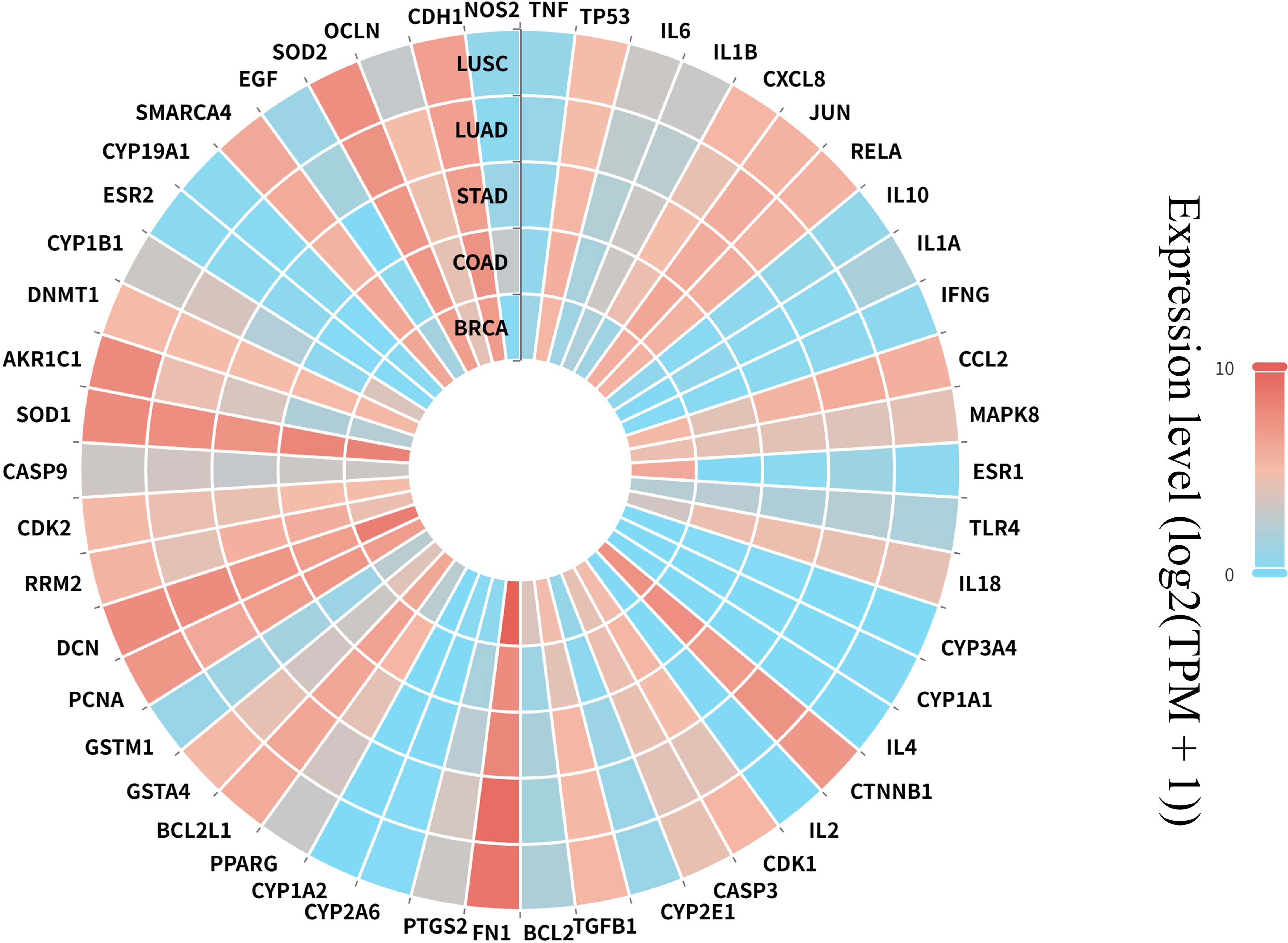

Supplement: SUPPLEMENTARY FIGURE 3 — Heatmap visualization of differential expression patterns for the 50 AFCs-Cancer core targets in five common cancers (LUSC, LUAD, COAD, BRCA, STAD). [file Image_3.JPEG]

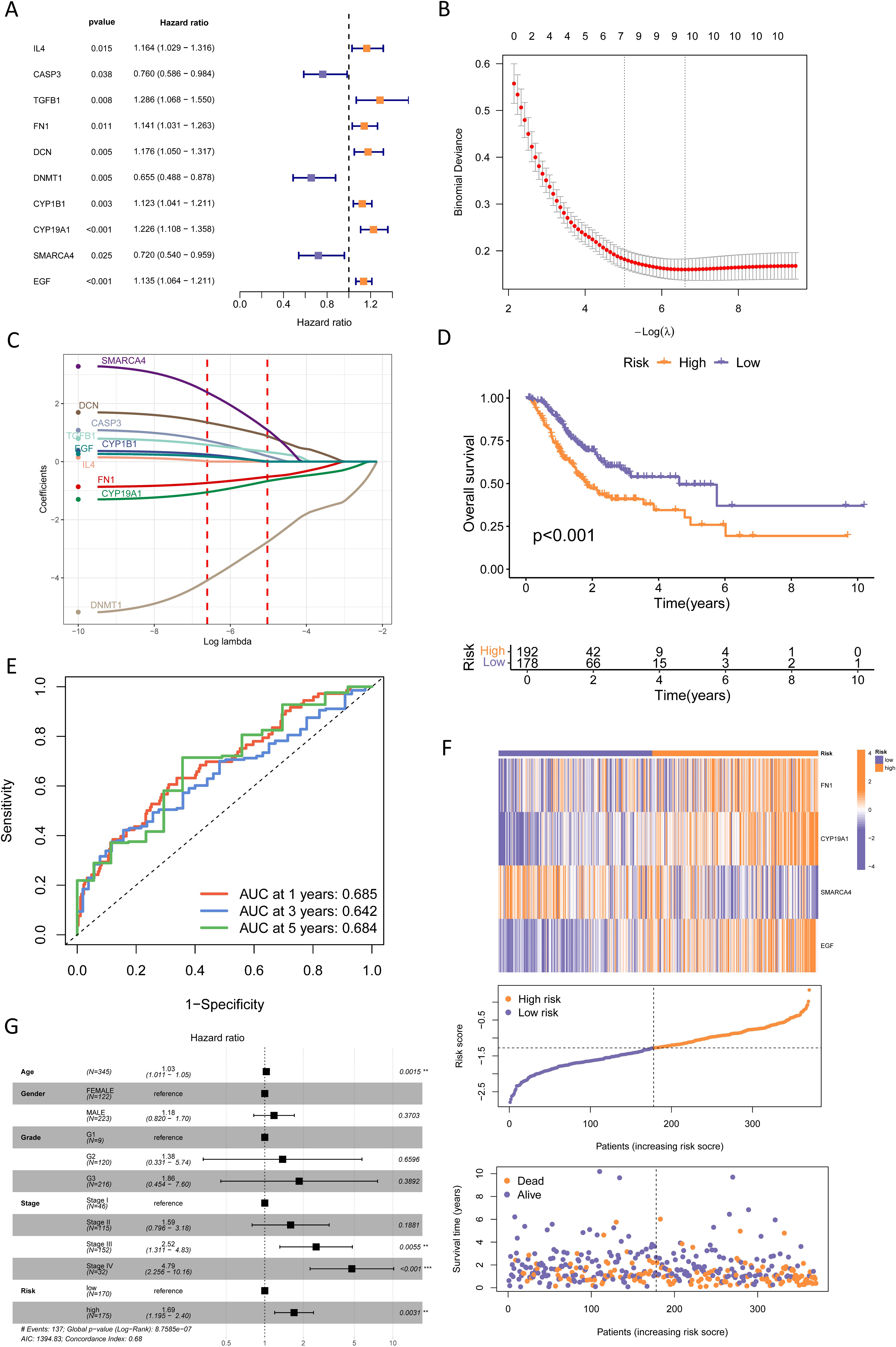

Supplement: SUPPLEMENTARY FIGURE 4 — Construction of a prognostic risk model for GC. (A) Univariate Cox regression analysis identified 10 prognostic genes (p < 0.05). (B) Coefficient profile plot generated from the Lasso regression analysis, with the optimal lambda (λ) value indicated by the right vertical dashed line. (C) Lasso regression analysis based on the 10 prognostic genes. (D) Kaplan–Meier survival analysis comparing high-risk and low-risk groups. (E) Time-dependent ROC curves of the prognostic model for 1-, 3-, and 5-year overall survival. (F) Visualization of the relationship between patient survival status and risk score, as well as the association between prognostic gene expression and risk score. The dashed line represents the cutoff between low-risk and high-risk groups. (G) Multivariate Cox regression analysis of clinical characteristics and risk groups. [file Image_4.JPEG]

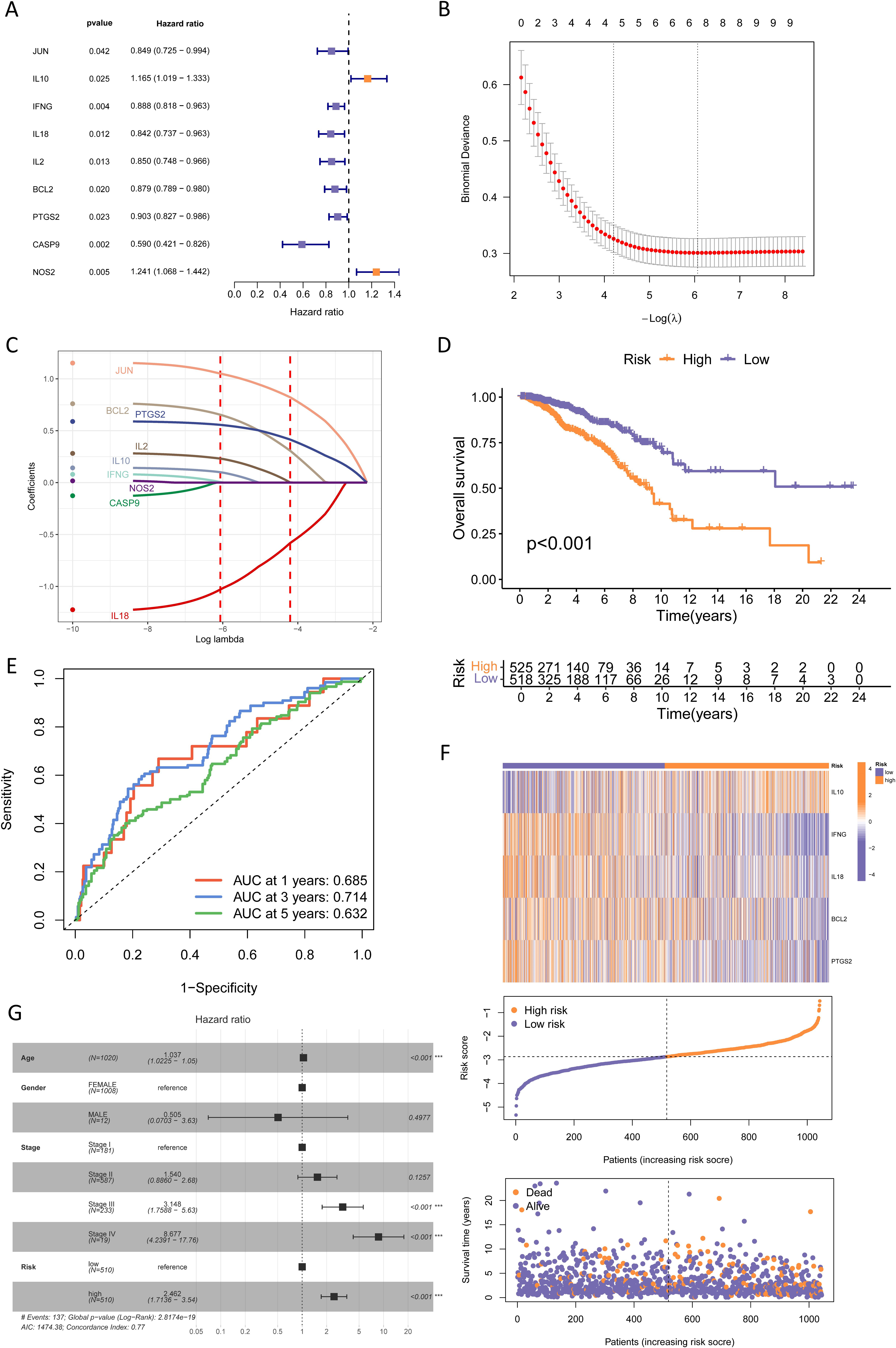

Supplement: SUPPLEMENTARY FIGURE 5 — Construction of a prognostic risk model for BRCA. (A) Univariate Cox regression analysis identified nine prognostic genes (p < 0.05). (B) Coefficient profile plot generated from the Lasso regression analysis, with the optimal lambda (λ) value indicated by the right vertical dashed line. (C) Lasso regression analysis based on the seven prognostic genes. (D) Kaplan–Meier survival analysis comparing high-risk and low-risk groups. (E) Time-dependent ROC curves of the prognostic model for 1-, 3-, and 5-year overall survival. (F) Visualization of the relationship between patient survival status and risk score, as well as the association between prognostic gene expression and risk score. The dashed line represents the cutoff between low-risk and high-risk groups. (G) Multivariate Cox regression analysis of clinical characteristics and risk groups. [file Image_5.JPEG]

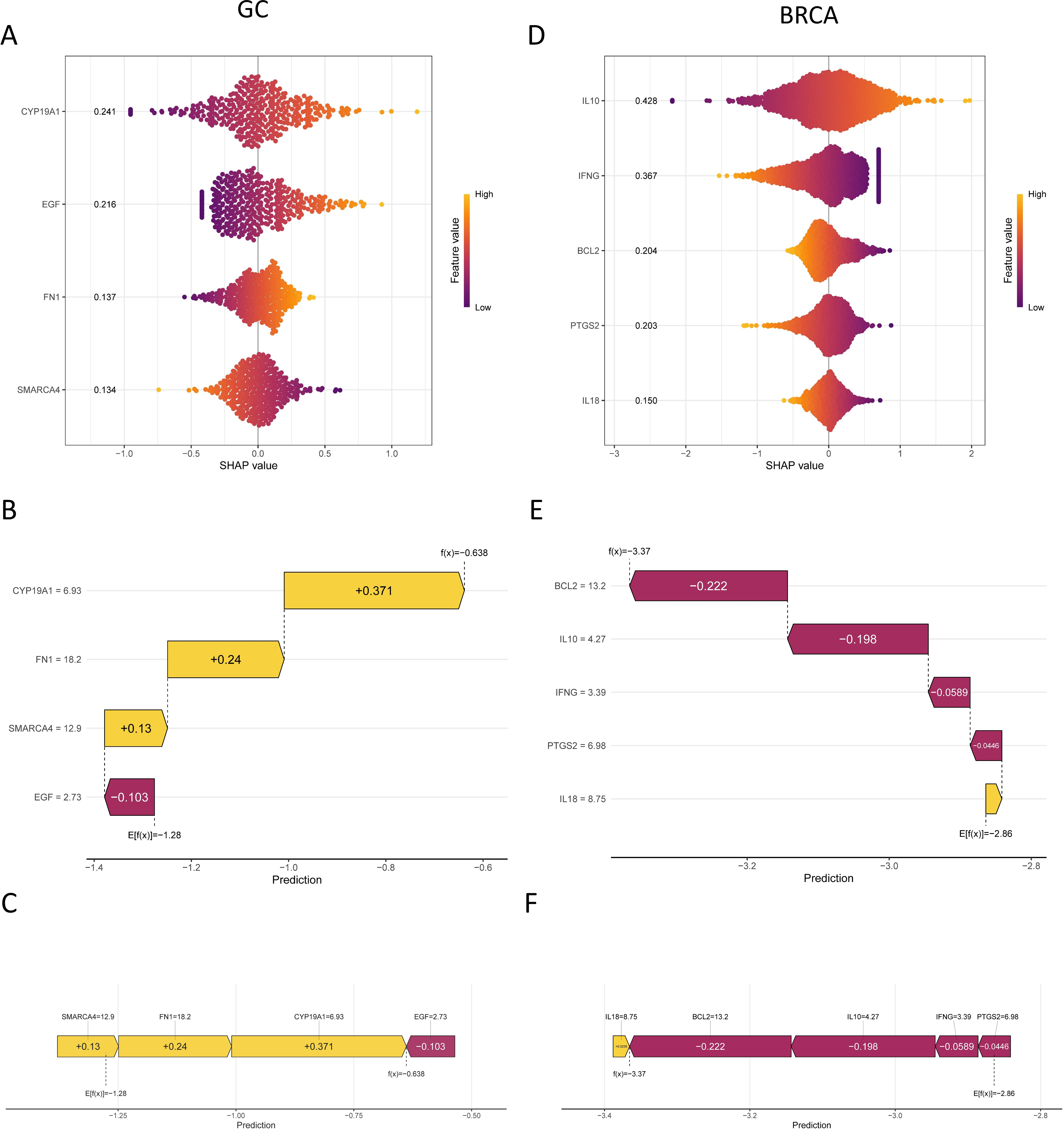

Supplement: SUPPLEMENTARY FIGURE 6 — SHAP values of prognostic genes in the prognostic models for gastric cancer and breast cancer. (A) SHAP summary plot for the gastric cancer model. (B) SHAP waterfall plot for the gastric cancer model. (C) SHAP force plot for the gastric cancer model. (D) SHAP summary plot for the breast cancer model. (E) SHAP waterfall plot for the breast cancer model. (F) SHAP force plot for the breast cancer model. [file Image_6.JPEG]

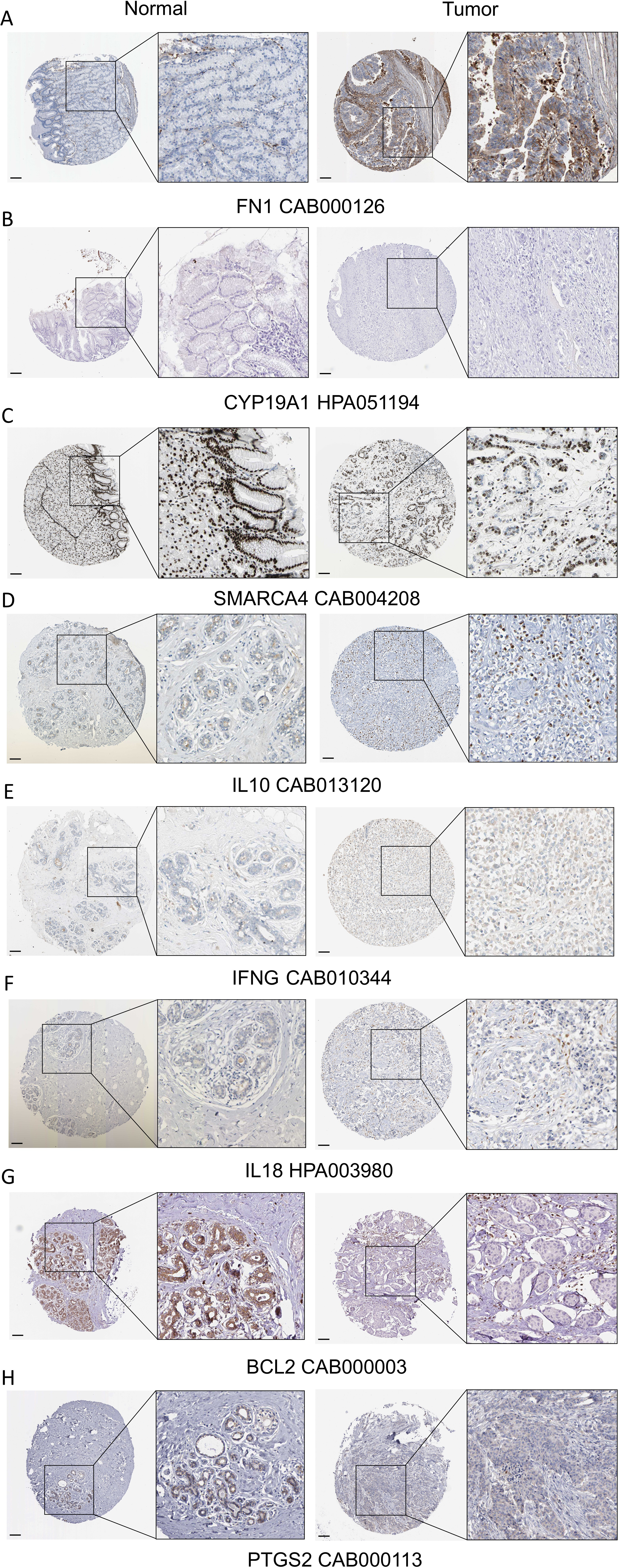

Supplement: SUPPLEMENTARY FIGURE 7 — Validation of prognostic gene expression in cancer tissues and matched normal tissues from GC and BRCA. (A–C) Expression levels of FN1, CYP19A1, and SMARCA4 in gastric cancer tissues and matched normal gastric tissues. (D–H) Expression levels of IL10, IFNG, IL18, BCL2, and PTGS2 in breast cancer tissues and matched normal breast tissues. [file Image_7.JPEG]

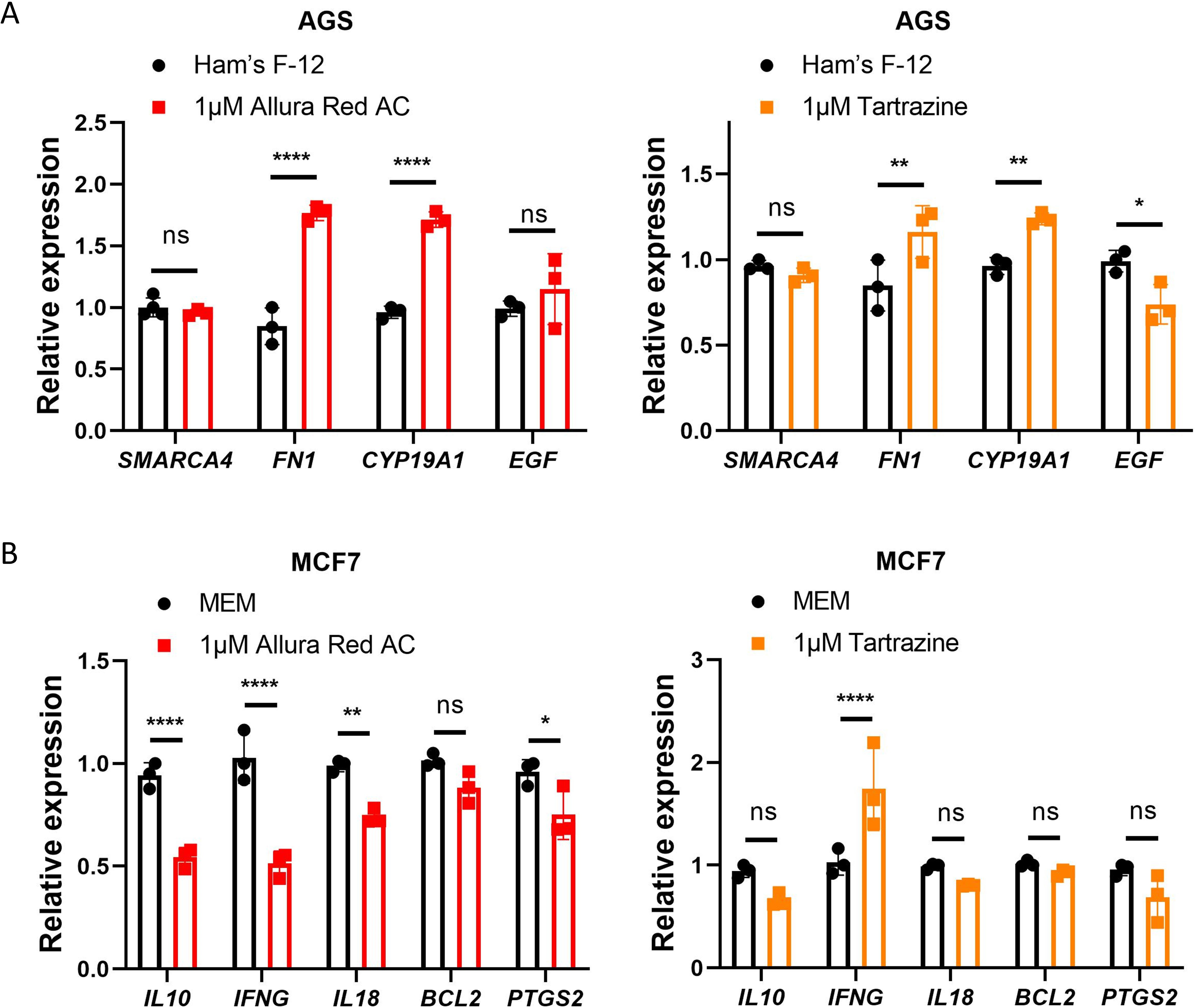

Supplement: SUPPLEMENTARY FIGURE 8 — qPCR validation of the effects of AFCs on the expression of AFCs-cancer prognostic targets in AGS cells and MCF-7 cells. Data are presented as mean ± SD. Statistical significance was determined by two-way ANOVA. *, p < 0.05; **, p < 0.01; ****, p < 0.0001; ns, not significant. [file Image_8.jpg]

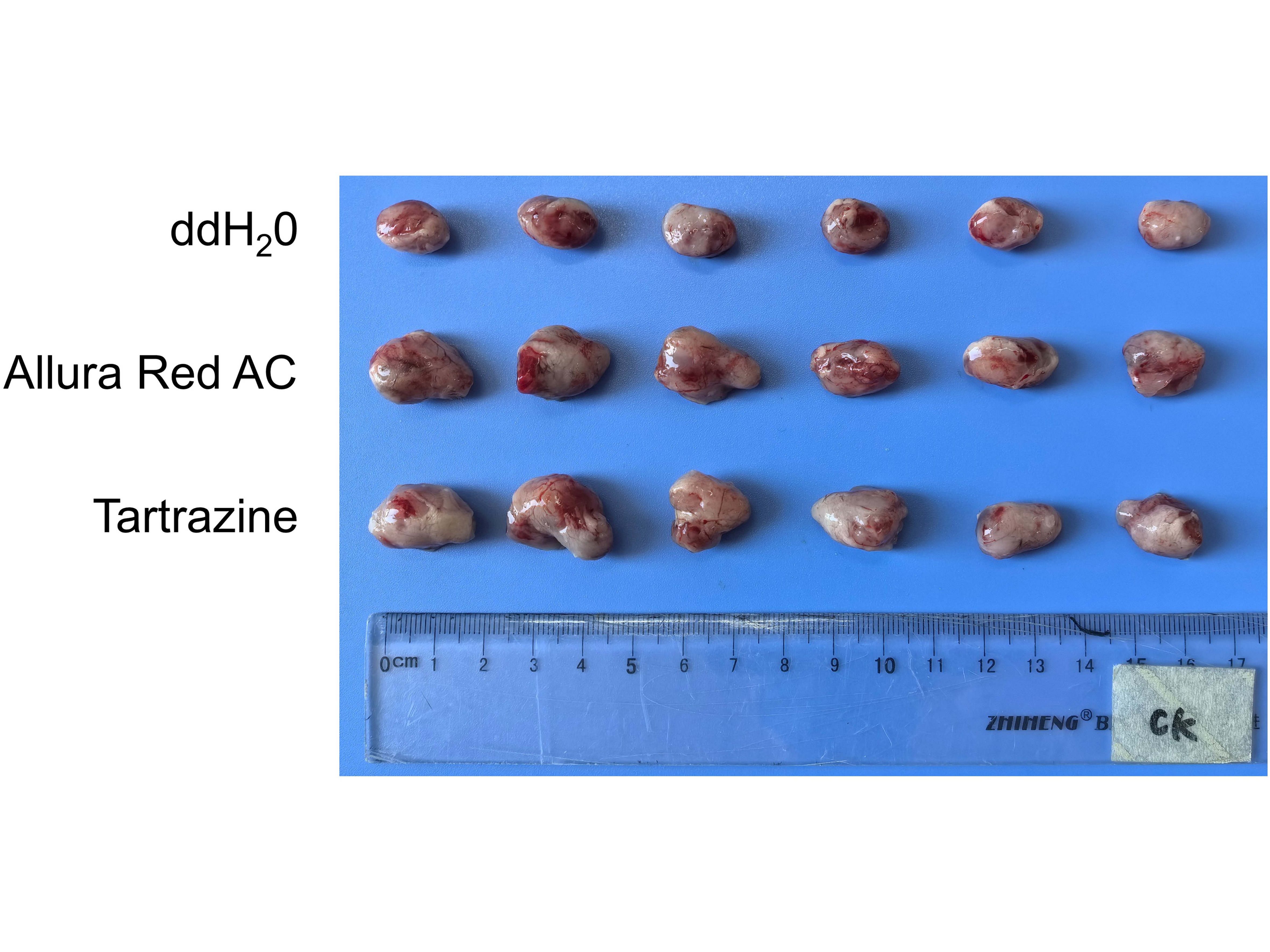

Supplement: SUPPLEMENTARY FIGURE 9 — Representative images of tumors collected on day 16 after LLC inoculation in C57BL/6 mice treated with AFCs (n = 6 per group). [file Image_9.JPEG]
