# Supplementary material for: A multi-omics approach elucidates the link between artificial food colorings and common cancers
Source: Front Nutr. 2026 Feb 5;13:1743416. doi: 10.3389/fnut.2026.1743416 (PMC12916573; doi:10.3389/fnut.2026.1743416)
Supplement: Supplementary file 10 [file Table_1.docx]

**Supplementary Table 1. The certification of color additives by FDA in Fiscal Year 2025.**

| FD&C number | Name | Pounds |
| --- | --- | --- |
| FD&C RED No. 40 | Allura Red AC | 8383764.08 |
| FD&C YELLOW No. 6 | Sunset Yellow FCF‌ | 5968465.24 |
| FD&C YELLOW No. 5 | Tartrazine | 4887106.96 |
| FD&C BLUE No. 1 | Brilliant Blue FCF | 1515246.32 |
| FD&C BLUE No. 2 | Indigo carmine | 465719.98 |
| FD&C RED No. 3 | Erythrosine | 156791.27 |
| FD&C GREEN No. 3 | Fast Green FCF | 13311.88 |

FDA, US Food and Drug Administration.
