# Supplementary material for: A multi-omics approach elucidates the link between artificial food colorings and common cancers
Source: Front Nutr. 2026 Feb 5;13:1743416. doi: 10.3389/fnut.2026.1743416 (PMC12916573; doi:10.3389/fnut.2026.1743416)
Supplement: Supplementary file 11 [file Table_2.docx]

**Supplementary Table 2. Approval status and ADI comparison of high-exposure AFCs in the US, EU, and China.**

| Common Name | US  (FDA) | EU (EFSA) | China (2760-2024) | International ADI (JECFA) (mg/kg bw) | ADI  (FDA) (mg/kg bw) | ADI (EFSA) (mg/kg bw) | ADI (China) (mg/kg bw) |
| --- | --- | --- | --- | --- | --- | --- | --- |
| Allura Red AC | Approved  (FD&C Red No. 40) | Approved (E129) | Approved | 0-7 | unpublished | 0-7 | unpublished |
| Sunset Yellow FCF‌ | Approved  (FD&C Yellow No. 6) | Approved (E110) | Approved | 0-4 | unpublished | 0-4 | unpublished |
| Tartrazine | Approved  (FD&C Yellow No. 5) | Approved (E102) | Approved | 0-10 | unpublished | 0-7.5 | unpublished |
| Brilliant Blue FCF‌ | Approved  (FD&C Blue No. 1) | Approved (E133) | Approved | 0-6 | unpublished | 0-6 | unpublished |

ADI, acceptable daily intake; US, The United States of America; EU, European Union; FDA, US Food and Drug Administration; EFSA, European Food Safety Authority; GB 2760-2014, National Food Safety Standard-Standard for Uses of Food Additives (GB 2760-2024); JECFA, Joint FAO/WHO Expert Committee on Food Additives.
