# Supplementary material for: A multi-omics approach elucidates the link between artificial food colorings and common cancers
Source: Front Nutr. 2026 Feb 5;13:1743416. doi: 10.3389/fnut.2026.1743416 (PMC12916573; doi:10.3389/fnut.2026.1743416)
Supplement: Supplementary file 12 [file Table_3.docx]

**Supplementary Table 3. The 50 AFCs–cancer core targets and supporting evidence from the literature.**

| Target | Title | PMID |
| --- | --- | --- |
| TNF | Tumour necrosis factor and cancer | 19343034 |
| TP53 | Mutational processes shape the landscape of TP53 mutations in human cancer | 30224644 |
| IL6 | Targeting the IL-6/JAK/STAT3 signalling axis in cancer | 29405201 |
| IL1B | Targeting interleukin-1β and inflammation in lung cancer | 35086565 |
| CXCL8 | The CXCL8-CXCR1/2 pathways in cancer | 27578214 |
| JUN | Oncofetal HLF transactivates c-Jun to promote hepatocellular carcinoma development and sorafenib resistance. | 31118247 |
| RELA | Negative regulation of RelA phosphorylation: emerging players and their roles in cancer. | 25438737 |
| IL10 | Interleukin-10 in cancer immunotherapy: from bench to bedside. | 37321942 |
| IL1A | IL-1α facilitates GSH synthesis to counteract oxidative stress in oral squamous cell carcinoma under glucose-deprivation | 38548217 |
| IFNG | Interferon-gamma: teammate or opponent in the tumour microenvironment? | 34155388 |
| CCL2 | The role of CCL2/CCR2 axis in cancer and inflammation: The next frontier in nanomedicine. | 38643840 |
| MAPK8 | MAPK8 mediates resistance to temozolomide and apoptosis of glioblastoma cells through MAPK signaling pathway. | 30119215 |
| ESR1 | ESR1 mutations and therapeutic resistance in metastatic breast cancer: progress and remaining challenges | 34621045 |
| TLR4 | Promotion of hepatocellular carcinoma by the intestinal microbiota and TLR4 | 22516259 |
| IL18 | The role of interleukin-18 in pancreatitis and pancreatic cancer. | 31753718 |
| CYP3A4 | Activation/Inactivation of Anticancer Drugs by CYP3A4: Influencing Factors for Personalized Cancer Therapy. | 36732076 |
| CYP1A1 | Aryl hydrocarbon receptor/cytochrome P450 1A1 pathway mediates breast cancer stem cells expansion through PTEN inhibition and beta-Catenin and Akt activation. | 28103884 |
| IL4 | IL4 Primes the Dynamics of Breast Cancer Progression via DUSP4 Inhibition. | 28400477 |
| CTNNB1 | ACLY facilitates colon cancer cell metastasis by CTNNB1. | 31511060 |
| IL2 | Reigniting hope in cancer treatment: the promise and pitfalls of IL-2 and IL-2R targeting strategies. | 37516849 |
| CDK1 | Deubiquitylase YOD1 regulates CDK1 stability and drives triple-negative breast cancer tumorigenesis. | 37667382 |
| CASP3 | Cleavage of CAD by caspase-3 determines the cancer cell fate during chemotherapy. | 40442064 |
| CYP2E1 | Identification of Cytochrome P450 2E1 as a Novel Target in Glioma and Development of Its Inhibitor as an Anti-Tumor Agent | 37283464 |
| TGFB1 | TGFB1/INHBA Homodimer/Nodal-SMAD2/3 Signaling Network: A Pivotal Molecular Target in PDAC Treatment. | 33429081 |
| BCL2 | BCL2: A promising cancer therapeutic target | 28647470 |
| FN1 | Src/FN1 pathway activation drives tumor cell cluster formation and metastasis in lung cancer: A promising therapeutic target. | 40632865 |
| PTGS2 | Improved Survival With Adjuvant Cyclooxygenase 2 Inhibition in PIK3CA-Activated Stage III Colon Cancer: CALGB/SWOG 80702 (Alliance). | 38889377 |
| CYP2A6 | Pretreatment with 8-methoxypsoralen, a potent human CYP2A6 inhibitor, strongly inhibits lung tumorigenesis induced by 4-(methylnitrosamino)-1-(3-pyridyl)-1-butanone in female A/J mice | 14633670 |
| CYP1A2 | CYP1A2 suppresses hepatocellular carcinoma through antagonizing HGF/MET signaling. | 33500715 |
| PPARG | Pparg signaling controls bladder cancer subtype and immune exclusion. | 34697317 |
| BCL2L1 | A selective BCL-X(L) PROTAC degrader achieves safe and potent antitumor activity. | 31792461 |
| GSTA4 | Glutathione S-transferase alpha 4 induction by activator protein 1 in colorectal cancer | 27065323 |
| GSTM1 | Exploring the impact of GSTM1 as a novel molecular determinant of survival in head and neck cancer patients of African descent | 39044272 |
| PCNA | PCNA: a silent housekeeper or a potential therapeutic target? | 24655521 |
| DCN | Decorin deficiency promotes epithelial-mesenchymal transition and colon cancer metastasis. | 33065248 |
| RRM2 | RRM2 Regulates Sensitivity to Sunitinib and PD-1 Blockade in Renal Cancer by Stabilizing ANXA1 and Activating the AKT Pathway. | 34319001 |
| CDK2 | CDK2-activated TRIM32 phosphorylation and nuclear translocation promotes radioresistance in triple-negative breast cancer. | 37734566 |
| CASP9 | Plumbagin engenders apoptosis in lung cancer cells via caspase-9 activation and targeting mitochondrial-mediated ROS induction. | 32034669 |
| SOD1 | Simultaneous Targeting of NQO1 and SOD1 Eradicates Breast Cancer Stem Cells via Mitochondrial Futile Redox Cycling. | 39264695 |
| AKR1C1 | Aldo-Keto Reductase AKR1C1-AKR1C4: Functions, Regulation, and Intervention for Anti-cancer Therapy. | 28352233 |
| DNMT1 | DNMT1: A key drug target in triple-negative breast cancer. | 32461152 |
| CYP1B1 | CYP1B1 and hormone-induced cancer. | 22561558 |
| ESR2 | ESR2 Drives Mesenchymal-to-Epithelial Transition in Triple-Negative Breast Cancer and Tumorigenesis In Vivo. | 35719919 |
| CYP19A1 | Targeting inhibition of prognosis-related lipid metabolism genes including CYP19A1 enhances immunotherapeutic response in colon cancer. | 37055842 |
| SMARCA4 | SMARCA4: Current status and future perspectives in non-small-cell lung cancer. | 36450331 |
| EGF | Histone Methyltransferase KMT2B Promotes Metastasis and Angiogenesis of Cervical Cancer by Upregulating EGF Expression. | 36594087 |
| SOD2 | MnSOD upregulation sustains the Warburg effect via mitochondrial ROS and AMPK-dependent signalling in cancer. | 25651975 |
| OCLN | DUSP3 regulates phosphorylation-mediated degradation of occludin and is required for maintaining epithelial tight junction | 35705979 |
| CDH1 | Hereditary Diffuse Gastric Cancer Syndrome: CDH1 Mutations and Beyond | 26182300 |
| NOS2 | NOS2 as an Emergent Player in Progression of Cancer | 28506076 |
